# Antiallergic drug desloratadine as a selective antagonist of 5HT_2A_ receptor ameliorates pathology of Alzheimer's disease model mice by improving microglial dysfunction

**DOI:** 10.1111/acel.13286

**Published:** 2020-12-24

**Authors:** Jian Lu, Chuzhao Zhang, Jianlu Lv, Xialin Zhu, Xingwu Jiang, Weiqiang Lu, Yin Lu, Zongxiang Tang, Jiaying Wang, Xu Shen

**Affiliations:** ^1^ Jiangsu Key Laboratory for Pharmacology and Safety Evaluation of Chinese Materia Medica and State Key Laboratory Cultivation Base for TCM Quality and Efficacy Nanjing University of Chinese Medicine Nanjing China; ^2^ Shanghai Key Laboratory of Regulatory Biology Institute of Biomedical Sciences and School of Life Sciences East China Normal University Shanghai China

**Keywords:** 5HT_2A_R, Alzheimer's disease, desloratadine, microglia, neuroinflammation

## Abstract

Alzheimer's disease (AD) is a progressively neurodegenerative disease characterized by cognitive deficits and alteration of personality and behavior. As yet, there is no efficient treatment for AD. 5HT_2A_ receptor (5HT_2A_R) is a subtype of 5HT_2_ receptor belonging to the serotonin receptor family, and its antagonists have been clinically used as antipsychotics to relieve psychopathy. Here, we discovered that clinically first‐line antiallergic drug desloratadine (DLT) functioned as a selective antagonist of 5HT_2A_R and efficiently ameliorated pathology of APP/PS1 mice. The underlying mechanism has been intensively investigated by assay against APP/PS1 mice with selective 5HT_2A_R knockdown in the brain treated by adeno‐associated virus (AAV)‐ePHP‐*si*‐*5HT_2A_R*. DLT reduced amyloid plaque deposition by promoting microglial Aβ phagocytosis and degradation, and ameliorated innate immune response by polarizing microglia to an anti‐inflammatory phenotype. It stimulated autophagy process and repressed neuroinflammation through 5HT_2A_R/cAMP/PKA/CREB/Sirt1 pathway, and activated glucocorticoid receptor (GR) nuclear translocation to upregulate the transcriptions of phagocytic receptors TLR2 and TLR4 in response to microglial phagocytosis stimulation. Together, our work has highly supported that 5HT_2A_R antagonism might be a promising therapeutic strategy for AD and highlighted the potential of DLT in the treatment of this disease.

## Graphical abstract

DLT as a 5HT_2A_R antagonist effectively improved cognitive impairment of APP/PS1 mice. Briefly, DLT reduced amyloid plaque deposition by promoting microglial Aβ phagocytosis and degradation, and ameliorated immune response by inhibiting translocation of NF‐κB. It stimulated autophagy process and repressed neuroinflammation through 5HT_2A_R/cAMP/PKA/CREB/Sirt1 pathway, and activated GR nuclear translocation to upregulate the expression of TLR2/4 in response to microglial phagocytosis stimulation.

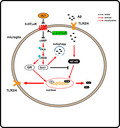

**Abbreviations**
5HT_2A_R: 5HT_2A_ receptor
AAV: adeno‐associated virus
AD: Alzheimer's disease
Aβ: amyloid‐β
CQ: Chloroquine
DLT: desloratadine
GPCR: G protein‐coupled receptor
GR: glucocorticoid receptor
H1R: Histamine 1 receptor
LTP: long‐term potentiation
NFTs: neurofibrillary tangles
SYP: synaptophysin
TLR2/4: toll‐like receptors 2 and 4

## 1 INTRODUCTION

Alzheimer's disease (AD) is a progressively neurodegenerative disease characterized by deficit of cognition and alteration of personality and behavior. Despite the enormous efforts in fighting against AD during the last three decades, there has been yet no effective medication to treat this disease (Brambilla, 2017). The histopathology of AD is mostly defined by the accumulation of amyloid‐β (Aβ) plaques and formation of neurofibrillary tangles (NFTs) in brains (Vijayraghavan et al., 2018). Extracellular amyloid plaques are mainly formed by Aβ aggregation, which is believed to be a key step in the pathogenesis of AD (Parhizkar et al., 2019). Data from preclinical and clinical studies have indicated that the unbalance between Aβ generation and clearance is tightly associated with AD pathogenesis, and the microglia‐mediated neuroinflammation induced by Aβ directly causes neuronal damage including neuron and synapse loss, which is one of the leading causes of AD progression (Zhong et al., 2019).

Microglia as the main immune cells in the brain participate in normal function and defense of central nervous system. Similar to periphery macrophages, microglia respond to micro‐environmental disturbance by drastically altering phenotypes and functions (Chen et al., 2014). In the pathogenesis of AD, microglia cluster around amyloid plaques and phagocytize the extracellular harmful proteins through phagocytic receptors (e.g., Toll‐like receptors, scavenger receptors, and TREM2) and further degrade them through lysosomal‐dependent ways including autophagy (Lee et al., 2018). At the same time, microglia provoke the conversion of a ‘resting’ type into an anti‐inflammatory phenotype involving homeostasis, regeneration, and neuroprotection (Lee et al., 2018). However, once the phagocytosis and degradation function of microglia is destroyed, excessive endogenous toxic proteins transform microglia from anti‐inflammatory phenotype to a pro‐inflammatory phenotype that is associated with inflammation response, neuronal damage, and death (Currais et al., 2014). Thus, it is suggested that improving microglial dysfunction to prevent microglia‐mediated inflammation should be a potential strategy for treating AD (Zhong et al., 2019).

Currently, several kinds of clinical drugs against AD are available (e.g., cholinesterase inhibitors and N‐methyl‐D‐aspartate receptor antagonists), but they can only relieve the pathological symptoms of the disease. As approximately 30%–50% of AD patients are accompanied by neuropsychiatric symptoms including depression and agitation, some antipsychotics are also commonly used as concomitant drugs for AD patients (Wilkins & Sambamoorthi, 2011). Although it was concluded that continued long‐term treatment with antipsychotics may reduce the rate of dementia in AD patients, the underlying mechanism is still unclear (Moraros et al., 2017).

5HT_2A_ receptor (5HT_2A_R) is a subtype of 5HT_2_ receptor belonging to the serotonin receptor family (Lippold & Dewey, 2017). As a type of G protein‐coupled receptor (GPCR) primarily coupling to Gαq signal transduction pathway, 5HT_2A_R expresses widely throughout the central nervous system and participates in varied brain functions, such as appetite control, thermoregulation, and sustained attention (Zhang & Stackman, 2015). Notably, 5HT_2A_R also functions potently in cognitive and noncognitive behaviors of AD and its inverse agonists and antagonists as antipsychotics ameliorate cognitive dysfunction and psychopathy. For example, 5HT_2A_R antagonist Pimavanserin was recently reported to present positive top‐line results by its Phase 3 HARMONY study at clinical trials against AD ([Internet] ACADIA Pharmaceuticals Announces Pivotal Phase 3 HARMONY Trial Stopped Early for Positive Efficacy as Pimavanserin Meets the Primary Endpoint in Patients with Dementia‐Related Psychosis. https://www.businesswire.com/news/home/20190909005286/en/ACADIA‐Pharmaceuticals‐Announces‐Pivotal‐Phase‐3‐HARMONY).

**FIGURE 1:**
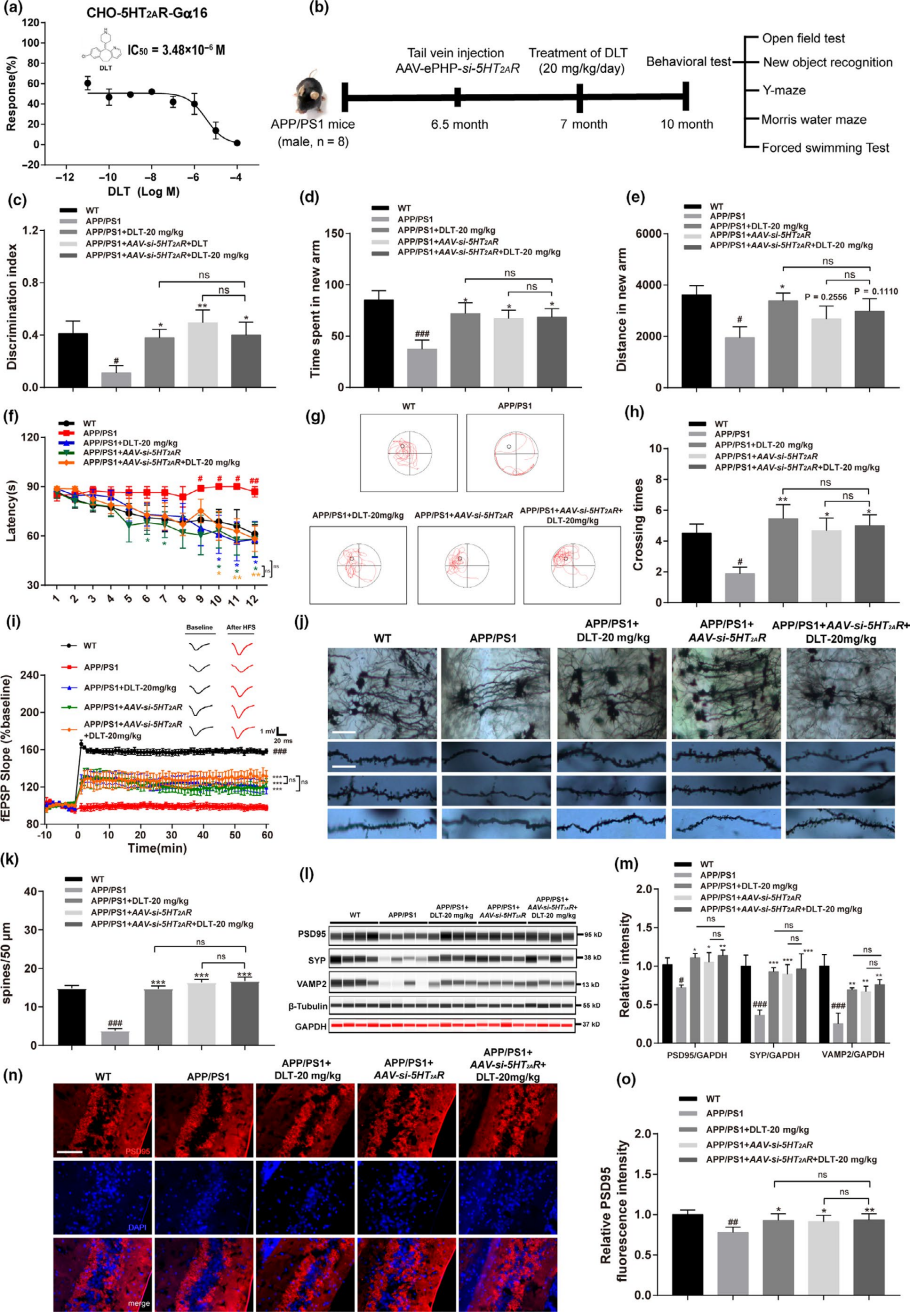
DLT treatment ameliorated the pathological behavior of APP/PS1 mice by antagonizing 5HT_2A_R. (a) Calcium assay results indicated that DLT dose‐dependently inhibited 5HT_2A_R in the presence of 10 μM of 5HT (IC_50_ = 3.48 μM) in CHO‐K1 cells. (b) Schedule of animal treatments and behavior tests, male WT, and APP/PS1 mice were used in the test. APP/PS1 mice were treated with DLT (20 mg/kg/day) at the age of seven months or injected with AAV‐ePHP‐*si*‐*5HT_2A_R* through tail vein injection at the age of six and a half months. The animal experiments were performed at the age of ten months of APP/PS1 mice (*n* = 8). (c) Assay result of new object recognition test indicated that treatment of DLT or AAV‐*si*‐*5HT_2A_R* ameliorated the short‐term working memory defect in APP/PS1 mice (*n* = 8). (d) Times spent in the target quadrant in probe trials (*n* = 8). (e) Results of Y‐maze test indicated that treatment of DLT or AAV‐*si*‐*5HT_2A_R* ameliorated the spatial working memory defect in APP/PS1 mice (*n* = 8). (f) Results of escape latency during platform trials indicated that treatment of DLT or AAV‐*si*‐*5HT_2A_R* ameliorated the learning performance defect in APP/PS1 mice (*n* = 8). (g) Representative tracing graphs of the probe trials. (h) Times of platform crossing in probe trials (*n* = 8). (i) Changes in fEPSP slope were recorded following high frequency stimulation (4 × 100 Hz) in hippocampal DG region of APP/PS1 mice, and treatment of DLT or AAV‐*si*‐*5HT_2A_R* effectively ameliorated LTP impairment in that region (brain slice, *n* = 2 brain slices per animal; animal, *n* = 2). (j) Golgi–Cox staining assay and (k) its quantification results indicated that treatment of DLT or AAV‐*si*‐*5HT_2A_R* reversed the spine deficiency of hippocampal neuron in APP/PS1 mice. Scale bar: 50 µm, 10 µm, respectively (brain slice, *n* = 3; cell, *n* = 10). (l) Jess assay and (m) its quantification results demonstrated that treatment of DLT or AAV‐*si*‐*5HT_2A_R* reversed the suppression of synapse‐related proteins, including PSD95, synaptophysin, and VAMP2 in the brains of APP/PS1 mice (*n* = 4). (n) Immunofluorescence assay against PSD95 and (o) its quantification demonstrated that treatment of DLT or AAV‐*si*‐*5HT_2A_R* efficiently reversed PSD95 protein deficiency in the hippocampal neurons of APP/PS1 mice (*n* = 4). Scale bar: 50 µm. All values were presented as mean ± SEM. Data were obtained from three independent experiments. ^##^ *p* < 0.01 ^###^ *p* < 0.001 compared with WT group by *t* test. *p < 0.05, **p < 0.01, ***p < 0.001 compared with APP/PS1 group by two‐way ANOVA

In the current work, we reported that clinically antiallergic drug desloratadine (DLT, Figure 1a) functioned as a selective antagonist of 5HT_2A_R and efficiently ameliorated AD pathology of APP/PS1 mice. The underlying mechanism has been intensively investigated by assay against the APP/PS1 mice with 5HT_2A_R knockdown selectively in the brain treated by adeno‐associated virus (AAV)‐ePHP‐*si*‐*5HT_2A_R*. Our work has strongly supported that 5HT_2A_R antagonism is a promising therapeutic strategy for AD and highlighted the potential of DLT in the treatment of this disease.

## 2 RESULTS

### 2.1 DLT was a selective antagonist of 5HT_2A_R

5HT_2A_R antagonist was randomly screened by Calcium flux assay in CHO‐K1 cells against the laboratory in‐house commercial FDA‐approved drug library, and antiallergic drug DLT was finally determined to be capable of inhibiting 5HT‐induced calcium flow activation by IC_50_ of 3.48 μM (Figure 1a).

In addition, results of calcium flow assays against primary neurons and microglia showed that DLT dose‐dependently inhibited calcium flow in neurons and microglia by IC_50_ at 9.177 μM and 0.3185 μM, respectively (Figure S1A,B). Moreover, 5HT_2A_R knockdown deprived DLT of its capability in regulating calcium flow in neurons and glia (Figure S1A,B). Thus, all results verified that DLT repressed calcium flow inhibition by its antagonism against 5HT_2A_R.

Finally, selectivity of DLT against 5HT_2A_R over other 5HTR subtypes and monoamine receptors was evaluated as depicted in Figure S10A–H. The results demonstrated that DLT was a selective antagonist of 5HT_2A_R.

### 2.2 DLT treatment ameliorated pathological behavior of APP/PS1 mice by antagonizing 5HT_2A_R

In an attempt to evaluate the capability of DLT in ameliorating memory and cognitive impairments of APP/PS1 mice, the models of new object recognition, Y‐maze, and Morris water maze were applied (for each test, *n* = 8).

In addition, to verify that DLT ameliorated the pathological behavior of APP/PS1 mice by antagonizing 5HT_2A_R, the assays were also performed against the APP/PS1 mice with selective 5HT_2A_R knockdown in the brain by injecting AAV‐ePHP‐*si*‐*5HT_2A_R*.

#### 2.2.1 New object recognition test

This test was performed to evaluate the short‐term working memory of mice. As indicated in Figure 1c, DLT‐ or AAV‐*si*‐*5HT_2A_R*‐treated APP/PS1 mice (APP/PS1+DLT or APP/PS1+AAV‐*si*‐*5HT_2A_R* mice) spent more time around the new object than vehicle‐treated APP/PS1 mice (APP/PS1 mice) (*F*_3,21_ = 3.583, *p* = 0.0310).

#### 2.2.2 Y‐maze test

This test was used to assess the spatial working memory mediated by hippocampus and prefrontal cortex. As indicated in Figure 1d,e, the time spent (*F*_3,21_ = 2.860, *p* = 0.0613) and distance travelled (*F*_3,21_ = 1.863, *p* = 0.1668) on new arm for APP/PS1+DLT or APP/PS1+AAV‐*si*‐*5HT_2A_R* mice were apparently ameliorated compared with those for APP/PS1 mice.

#### 2.2.3 Morris water maze test

This test was used to assess spatial learning and long‐term memory of mice. As indicated in Figure 1f, APP/PS1 + DLT or APP/PS1 + AAV‐*si*‐*5HT_2A_R* mice exhibited a decrease in the time required to reach the platform compared with APP/PS1 mice. This result thus indicated the improvement of the learning impairment of APP/PS1 + DLT or APP/PS1 + AAV‐*si*‐*5HT_2A_R* mice compared with APP/PS1 mice (*F*_3,336_ = 14.96, *p* < 0.0001). In addition, during the test in thirteenth day, APP/PS1 + DLT or APP/PS1 + AAV‐*si*‐*5HT_2A_R* mice spent more time staying in the target quadrant (*F*_3,21_ = 1.501, *p* = 0.2434) and crossed the target quadrant (*F*_3,21_ = 3.157, *p* = 0.0461) more frequently compared with APP/PS1 mice (Figure 1g,h). There was no difference in swimming speed among the mice (Figure S3C) (*F*_3,21_ = 1.094, *p* = 0.3736).

Notably, no significant difference was observed in the amelioration of any behavior tests (New object recognition test, Y‐maze test, and Morris water maze test) between DLT‐treated AAV‐*si*‐*5HT_2A_R*‐injected APP/PS1 mice (APP/PS1 + AAV‐*si*‐*5HT_2A_R*+DLT mice) and vehicle‐treated AAV‐*si*‐*5HT_2A_R*‐injected APP/PS1 mice (APP/PS1 + AAV‐*si*‐*5HT_2A_R* mice).

Taken together, all results demonstrated that DLT ameliorated pathological behavior of APP/PS1 mice by antagonizing 5HT_2A_R.

### 2.3 DLT treatment ameliorated synaptic plasticity and integrity in the hippocampus of APP/PS1 mice by antagonizing 5HT_2A_R

Given the potency of synapse in cognition and mental activity (Moriguchi et al., 2018), we inspected the potential of DLT in ameliorating synapse plasticity and integrity in the hippocampus of APP/PS1 mice.

#### 2.3.1 DLT treatment ameliorated LTP in APP/PS1 mice by antagonizing 5HT_2A_R

The long‐term potentiation (LTP) in hippocampal DG region was examined to evaluate synapse plasticity in APP/PS1 mice. As indicated in Figure 1i, DLT or AAV‐*si*‐*5HT_2A_R* treatment effectively improved LTP induction and maintenance in hippocampal DG region of APP/PS1 mice (*F*_3,1400_ = 261.5, *p* < 0.0001).

#### 2.3.2 DLT ameliorated synaptic integrity in APP/PS1 mice by antagonizing 5HT_2A_R

In addition, Golgi‐Cox staining assay was also performed to detect synapse integrity of hippocampal neurons of mice. As shown in Figure 1j,k, DLT or AAV‐*si*‐*5HT_2A_R* treatment obviously reversed the spine deficiency of hippocampal neurons in APP/PS1 mice (*F*_3,27_ = 89.82, *p* < 0.0001).

In addition, as shown in Figure 1l,m and Figure S3E, DLT or AAV‐*si*‐*5HT_2A_R* treatment increased the protein levels of synaptic associated proteins PSD95, VAMP2, and synaptophysin (SYP) (*F*_4,45_ = 13.53, *p* < 0.0001) (*F*_4,45_ = 17.76, *p* < 0.0001) in the brains of APP/PS1 mice. Moreover, synaptic marker protein PSD95 was also detected by immunofluorescence assay for its crucial function in neurotransmission and synaptic plasticity. As shown in Figure 1n,o, DLT or AAV‐*si*‐*5HT_2A_R* treatment efficiently reversed PSD95 protein deficiency in the hippocampal neuron of APP/PS1 mice (*F*_3,9_ = 4.749, *p* = 0.0299).

Notably, no significant difference was observed in the amelioration of synaptic plasticity and integrity between APP/PS1 + AAV‐*si*‐*5HT_2A_R*+DLT mice and APP/PS1 + AAV‐*si*‐*5HT_2A_R* mice.

Thus, all abovementioned results demonstrated that DLT treatment protected against the loss of synaptic plasticity and integrity in APP/PS1 mice by antagonizing 5HT_2A_R.

### 2.4 DLT promoted microglial phagocytosis of Aβ in the hippocampus of APP/PS1 mice through 5HT_2A_R‐mediated TLR2/4 upregulation

**FIGURE 2:**
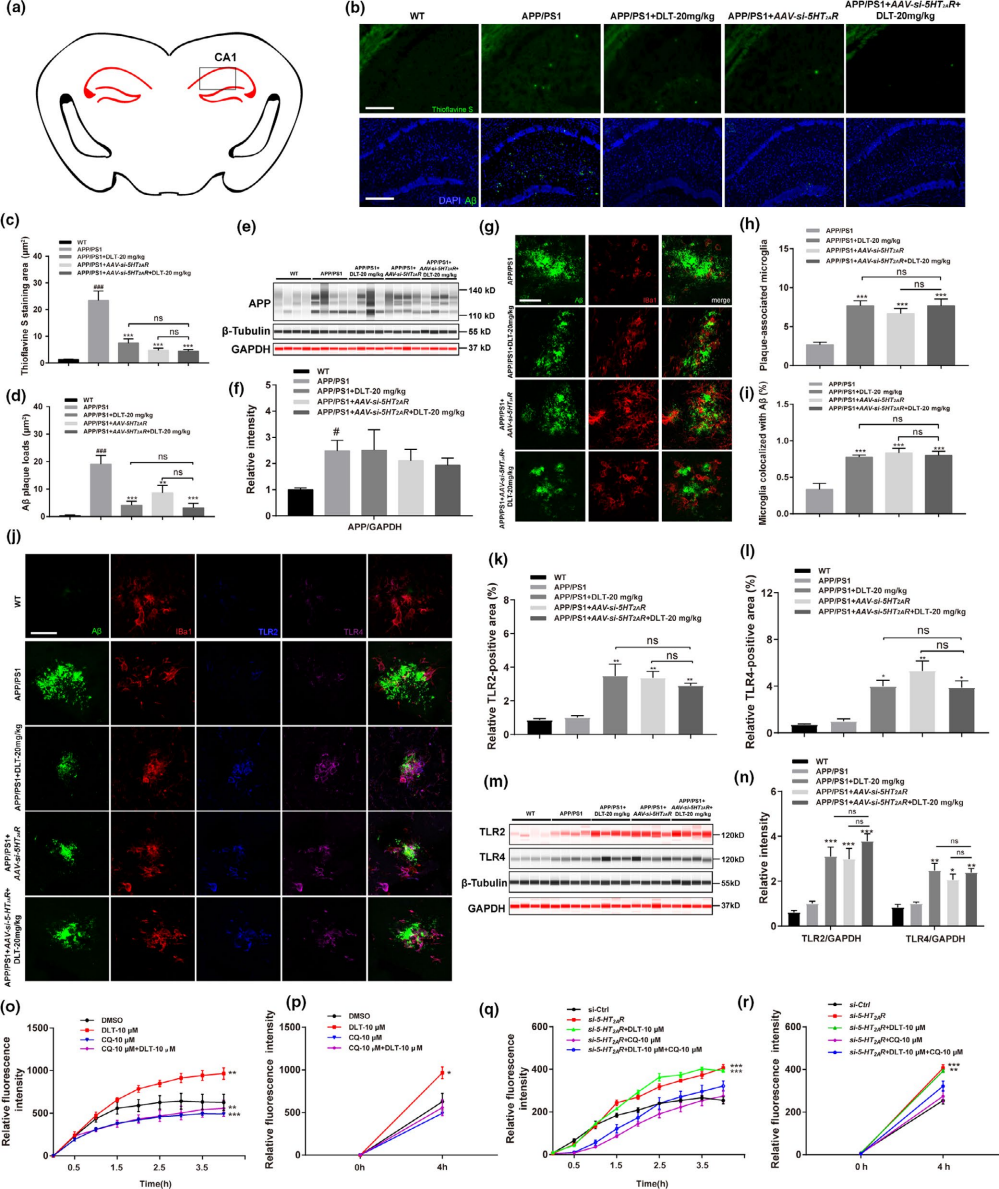
DLT promoted microglial phagocytosis of Aβ in the hippocampus of APP/PS1 mice through 5‐HT_2A_R‐mediated TLR2/4 upregulation. (a) Levels of senile plaque and Aβ plaque in the hippocampus (CA1 region) of APP/PS1 mice were detected. (b) Thioflavin S staining assay and (c) its quantification results demonstrated that treatment of DLT or AAV‐*si*‐*5HT_2A_R* reduced level of senile plaque in the hippocampus CA1 region of APP/PS1 mice (*n* = 4). Scale bar: 100 µm. (b) Immunofluorescence assay and (d) its quantification results demonstrated that treatment of DLT or AAV‐*si*‐*5HT_2A_R* reduced Aβ plaque in the hippocampus CA1 region of APP/PS1 mice (*n* = 4). Scale bar: 100 µm. (e) Jess assay and (f) its quantification results demonstrated that treatment of DLT or AAV‐*si*‐*5HT_2A_R* rendered no influences on APP protein level in the brains of APP/PS1 mice (*n* = 4). (g‐i) Microglia in the hippocampus (CA1 region) of APP/PS1 mice were identified by IBa1 antibody. (g) Immunofluorescence assay and (h, i) its quantification results demonstrated that treatment of DLT or AAV‐*si*‐*5HT_2A_R* increased the (h) number of plaque‐associated microglia and (i) ratio of microglia colocalized with Aβ in the hippocampus of APP/PS1 mice (*n* = 4). Scale bar: 10 µm. (j‐l) Levels of TLR2/4 in microglia of hippocampus (CA1 region) in APP/PS1 mice were detected by immunofluorescence assay. (j) Immunofluorescence assay and (k, l) its quantification results demonstrated that treatment of DLT or AAV‐*si*‐*5HT_2A_R* increased TLR2/4‐positive area in microglia of APP/PS1 mice (*n* = 4). Scale bar: 10 µm. (m) Jess assay and (n) its quantification results demonstrated that treatment of DLT or AAV‐*si*‐*5HT_2A_R* increased TLR2/4 protein levels in the brains of APP/PS1 mice (*n* = 4). (o‐r) Live cell imaging assay was performed to detect the microglial phagocytosis process of o‐Aβ_42_. (o, q) Real‐time detection of live cell imaging assay and (p, r) its quantification (levels of Aβ phagocytosis within four hours) results demonstrated that co‐treatment of TLR inhibitor CQ effectively abolished the phagocytosis effect induced by treatment of DLT or *si*‐*5HT_2A_R* (*n* = 3). All values were presented as mean ± SEM. ^#^ *p* < 0.05, ^##^ *p* < 0.01, ^###^ *p* < 0.001 compared with WT group by *t* test, *p < 0.05, **p < 0.01, ***p < 0.001 compared with APP/PS1 group by two‐way ANOVA

Considering that hippocampus as an essential brain region for declarative memory and cognition is tightly related to the onset and development of AD and CA1 region as the main area of hippocampal uptake of serotonin is much vulnerable to damage by inflammation and oxidative stress (Teixeira et al., 2018; Wang, Wang, et al., 2014), CA1 region should function potently in 5HT‐mediated hippocampus function in AD progress. As such, the hippocampal CA1 region (Figure 2a) was here selected as the main area for investigating the mechanism underlying the regulation of 5HT_2A_R antagonism against AD.

#### 2.4.1 DLT reduced senile plaque and Aβ plaque levels in CA1 region of hippocampus in APP/PS1 mice by antagonizing 5HT_2A_R

Given that senile plaque is a main hallmark of AD, levels of senile plaque and Aβ plaque (including Aβ_37_, Aβ_38_, Aβ_39_, Aβ_40_, and Aβ_42_) in CA1 region of hippocampus of APP/PS1 mice were detected by Thioflavin S staining and immunofluorescence assay. As shown in Figure 2b‐d, DLT or AAV‐*si*‐*5HT_2A_R* treatment reduced the levels of senile plaque (*F*_3,9_ = 31.50, *p* < 0.0001) and Aβ plaque (*F*_3,6_ = 27.22, *p* = 0.0007). Notably, no significant difference was determined in the levels of senile plaque and Aβ plaque between APP/PS1 + AAV‐*si*‐*5HT_2A_R*+DLT mice and APP/PS1 + AAV‐*si*‐*5HT_2A_R* mice.

Thus, all abovementioned results indicated that DLT reduced levels of senile plaque and Aβ plaque in CA1 region of hippocampus in APP/PS1 mice by antagonizing *5HT_2A_R*.

#### 2.4.2 DLT suppressed Aβ level independent of amyloidogenic pathway

Given that *5HT_2A_R* was ever reported to participate in the regulation of amyloid precursor protein (APP) ectodomain secretion and Aβ generation (Nitsch et al., 1996), we investigated whether DLT reduced Aβ level involving APP protein suppression by Jess assay. As indicated in Figure 2e,f and Figure S4A, DLT or AAV‐*si*‐*5HT_2A_R* treatment rendered no influences on APP protein level in the brains of APP/PS1 mice (*F*_4,15_ = 1.804, *p* = 0.1806) (*F*_4,15_ = 1.400, *p* = 0.2815). Moreover, DLT or AAV‐*si*‐*5HT_2A_R* treatment has no impacts on BACE1, sAPPβ, or p‐APP (*F*_3,36_ = 1.779, *p* = 0.1686) (*F*_3,36_ = 7.470, *p* = 0.0005) in the brains of APP/PS1 mice (Figure S4B‐D), thereby indicating that DLT suppressed Aβ level independent of amyloidogenic pathway.

#### 2.4.3 DLT promoted microglial phagocytosis of Aβ by antagonizing 5HT_2A_R

In AD brain, over‐aggregated Aβ is phagocytized by microglia and then degraded by intracellular auto‐degradation procedures such as autophagy (Lee et al., 2018). With these facts, we at first inspected the regulation of DLT against microglial phagocytosis of Aβ in the hippocampus of the mice.

In the assay, coronal sections of brain from APP/PS1 mice were stained with Aβ and IBa1 antibodies, microglia were identified by IBa1 antibody and the number of IBa1‐positive microglia in the vicinity of an Aβ plaque was quantified. As indicated in Figure 2g–i, DLT or AAV‐*si*‐*5HT_2A_R* treatment increased the number of plaque‐associated microglia (*F*_3,6_ = 18.80, *p* = 0.019) and the ratio of microglia colocalized with Aβ (*F*_3,6_ = 14.84, *p* = 0.0006) in the hippocampus of APP/PS1 mice. Notably, no significant difference was found in the regulation of either of these two items between APP/PS1 + AAV‐si‐*5HT_2A_R*+DLT mice and APP/PS1 + AAV‐*si*‐*5HT_2A_R* mice. Thus, all results demonstrated that DLT promoted microglial phagocytosis of Aβ by antagonizing 5HT_2A_R.

#### 2.4.4 DLT treatment promoted microglial phagocytosis of Aβ through 5HT_2A_R‐mediated TLR2/4 upregulation

Given that 5HT_2A_R antagonist was ever reported to regulate phagocytosis‐related receptors Toll‐like receptor 2 and 4 (TLR2/4) (Hung et al., 2016), we next inspected whether DLT or AAV‐*si*‐*5HT_2A_R* treatment promoted microglial phagocytosis of Aβ through TLR2/4 signaling by antagonizing 5HT_2A_R. As shown in Figure 2j–n and Figure S5A, immunofluorescence and Jess assays results indicated that DLT or AAV‐*si*‐*5HT_2A_R* treatment enhanced TLR2/4‐positive area (*F*_3,6_ = 11.48, *p* = 0.0067) (*F*_3,6_ = 9.864, *p* = 0.0099) in microglia, and protein levels (*F*_3,24_ = 19.04, *p* < 0.0001) (*F*_3,24_ = 18.66, *p* < 0.0001) in the brains of APP/PS1 mice. Notably, there was no significant difference in TLR2/4 regulation between APP/PS1 + AAV‐*si*‐*5HT_2A_R*+DLT mice and APP/PS1 + AAV‐*si*‐*5HT_2A_R* mice, thus demonstrating that DLT treatment upregulated TLR2/4 signaling by antagonizing 5HT_2A_R.

Moreover, as shown in Figure 2o–r, treatment of DLT or *si*‐*5HT_2A_R* enhanced FAM‐o‐Aβ_42_ level in microglia, but co‐treatment of TLRs inhibitor Chloroquine (CQ) (Zhu et al., 2012) with DLT or *si*‐*5HT_2A_R* effectively abolished such an enhancement (*F*_3,32_ = 2.768, *p* = 0.0583) (*F*_4,40_ = 1.329, *p* = 0.2758). Therefore, all results demonstrated that TLR2/4 signaling was responsible for DLT or AAV‐*si*‐*5HT_2A_R*‐mediated promotion on microglial phagocytosis of Aβ.

### 2.5 DLT treatment promoted microglial Aβ clearance in the hippocampus of APP/PS1 mice through 5HT_2A_R‐mediated autophagy stimulation

#### 2.5.1 DLT treatment stimulated microglial autophagy in the hippocampus of APP/PS1 mice by antagonizing 5HT_2A_R

To investigate the mechanism underlying the regulation of DLT against intracellular Aβ degradation in microglia, autophagy‐related study was addressed because autophagy as a potent auto‐degradation process plays a key role in Aβ clearance (Shin et al., 2014). In the assay, we investigated the regulation of DLT against autophagy by immunofluorescence imaging toward autophagy marker protein LC3.

**FIGURE 3:**
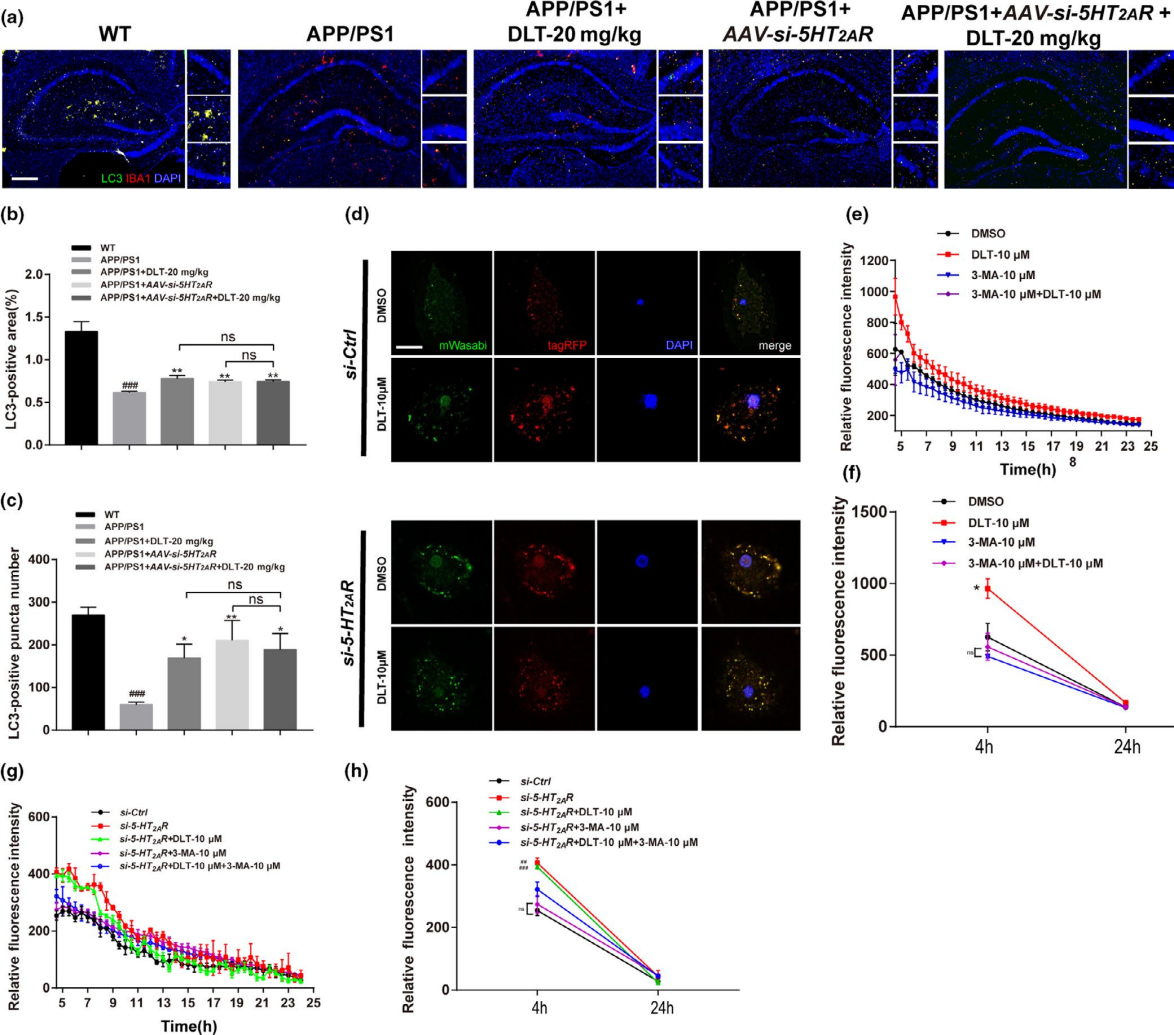
DLT treatment promoted microglial Aβ clearance in the hippocampus of APP/PS1 mice through 5‐HT_2A_R‐mediated autophagy stimulation. (a) Immunofluorescence assay and (b, c) its quantification results demonstrated that treatment of DLT or AAV‐*si*‐*5HT_2A_R* increased the number and area of LC3 positive puncta in the hippocampus of APP/PS1 mice (*n* = 4). Scale bar: 100 µm. (d) An overexpression vector encoding mTagRFP‐mWasabi‐LC3 was applied in primary microglia to verify the promotion of DLT on autophagic flux. The green and red puncta indicated mWasabi‐LC3 and autolysosomes, respectively, and merged puncta (yellow) of green and red fluorescent signals indicated autophagosomes. The results of autophagic flux demonstrated that both treatment of DLT and *si*‐*5HT_2A_R* stimulated autophagy in primary microglia (*n* = 4). Scale bar: 10 µm. (e, g) Real‐time detection results of live cell imaging assay and (f, h) its quantification (levels of Aβ degradation within twenty hours) demonstrated that co‐treatment of autophagy inhibitor 3‐MA effectively abolished the promotion of Aβ clearance induced by DLT or *si*‐*5HT_2A_R* treatment (*n* = 3). All values were presented as mean ± SEM. For animal tissue assays, ^###^ *p* < 0.001 compared with WT group by *t* test, *p < 0.05, **p < 0.01, ***p < 0.001 compared with APP/PS1 group by two‐way ANOVA. For cell assays, *p < 0.05 compared with DMSO or *si*‐*Ctrl* by one‐way ANOVA

As shown in Figure 3a–c, DLT or AAV‐*si*‐*5HT_2A_R* treatment enhanced the area (*F*_3,9_ = 8.792, *p* = 0.0049) and number of LC3‐positive puncta (*F*_3,9_ = 5.186, *p* = 0.0236) in the hippocampus of APP/PS1 mice. Notably, there was no significant difference in the regulation of LC3 between APP/PS1 + AAV‐*si*‐*5HT_2A_R* + DLT mice and APP/PS1 + AAV‐*si*‐*5HT_2A_R* mice.

Moreover, an overexpression vector encoding mTagRFP‐mWasabi‐LC3 was applied in primary microglia to further verify the promotion of DLT on autophagy (Zhou et al., 2012). As shown in Figure 3d, DLT or *si*‐*5HT_2A_R* treatment efficiently increased the amounts of autophagosomes and autolysosomes, thus indicating that both DLT and *si*‐*5HT_2A_R* stimulated autophagy in microglia.

#### 2.5.2 DLT promoted microglial Aβ clearance by stimulating autophagy

In addition, as shown in Figure E‐H, co‐treatment of autophagy inhibitor 3‐MA (Wang, Xu, et al., 2014) abolished the activity of DLT or *si*‐*5HT_2A_R* treatment in promoting Aβ clearance in microglia (*F*_4,164_ = 3.570, *p* = 0.0144) (*F*_4,195_ = 1.744, *p* = 0.1418), further indicating that DLT promoted Aβ clearance in microglia by stimulating autophagy.

Together, all results demonstrated that DLT treatment promoted microglial Aβ clearance in the hippocampus of APP/PS1 mice through stimulation of autophagy by antagonizing 5HT_2A_R.

### 2.6 DLT treatment repressed neuroinflammation in APP/PS1 mice by antagonizing 5HT_2A_R

Given that neuroinflammation induced by plaque‐associated microglia is tightly linked to AD progression, we detected the potential of DLT treatment in suppressing neuroinflammation.

#### 2.6.1 DLT treatment repressed neuroinflammation in the brains of APP/PS1 mice by antagonizing 5HT_2A_R

**FIGURE 4:**
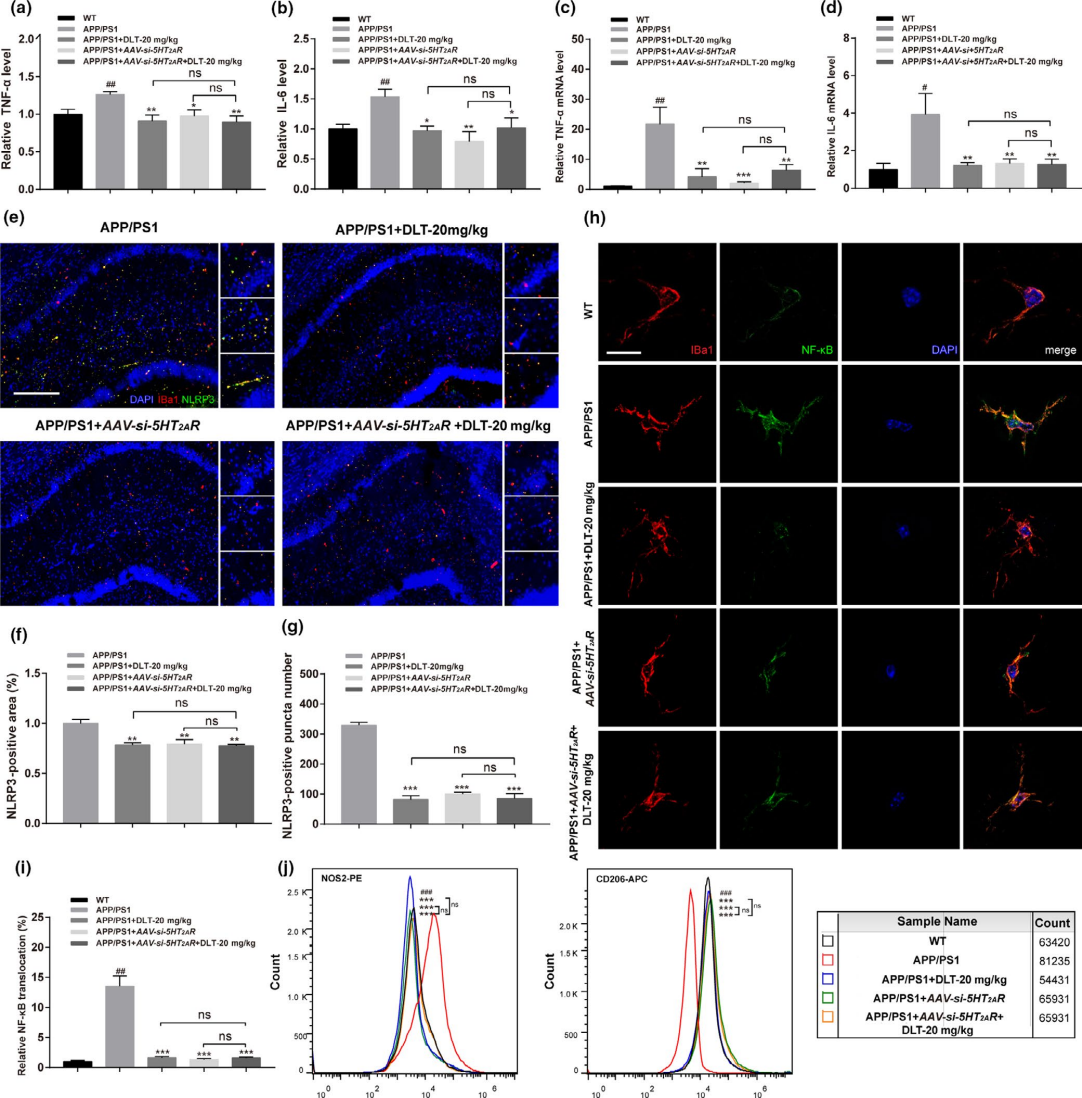
DLT treatment repressed microglial neuroinflammation in APP/PS1 mice by antagonizing 5‐HT_2A_R. (a, b) ELISA results demonstrated that treatment of DLT or AAV‐*si*‐*5HT_2A_R* reduced the protein levels of TNF‐α and IL‐6 in the brains of APP/PS1 mice (*n* = 5). (c, d) RT‐PCR results demonstrated that treatment of DLT or AAV‐*si*‐*5HT_2A_R* reduced the mRNA levels of TNF‐α and IL‐6 in the brains of APP/PS1 mice (*n* = 5). (e) Immunofluorescence assay and (f, g) its quantification results demonstrated that treatment of DLT or AAV‐*si*‐*5HT_2A_R* increased the area and number of NLRP3‐positive puncta in the hippocampus (CA1 region) of APP/PS1 (*n* = 4). Scale bar: 100 µm. (h) Immunofluorescence assay and (i) its quantification results demonstrated that treatment of DLT and AAV‐*si*‐*5HT_2A_R* suppressed the nuclear translocation rate of NF‐κB in hippocampal microglia of APP/PS1 (brain slice, *n* = 4; cell, *n* = 12). Scale bar: 10 µm. (j) Flow cytometry was performed to detect the M1/M2 microglial polarization in the brain of APP/PS1, and NOS2‐PE antibody and CD206‐APC antibody were used to mark M1 and M2 microglia, respectively. The results of flow cytometry showed that treatment of DLT or AAV‐*si*‐*5HT_2A_R* promoted the M1 to M2 microglial polarization in the brain of APP/PS1 mice (*n* = 2; sample size = 4). All values were presented as mean ± SEM. ^#^ *p* < 0.05, ^##^ *p* < 0.01 compared with WT group by *t* test. *p < 0.05, **p < 0.01, ***p < 0.001 compared with APP/PS1 group by two‐way ANOVA

ELISA and RT‐PCR assay results demonstrated that the protein and mRNA levels of pro‐inflammatory cytokines (TNF‐α (*F*_3,15_ = 5.745, *p* < 0.008) (*F*_3,15_ = 7.064, *p* = 0.0035) and IL‐6 (*F*_3,15_ = 4.334, *p* = 0.0275) (*F*_3,15_ = 4.864, *p* = 0.0147)) and anti‐inflammatory cytokines (IL‐4 (*F*_3,12_ = 13.36, *p* = 0.0004) (*F*_3,15_ = 3.126, *p* = 0.0531) and IL‐10 (*F*_3,12_ = 9.276, *p* = 0.0019) (*F*_3,15_ = 2.522, *p* = 0.0972)) were, respectively, decreased (Figure 4a–d) and increased (Figure S6A–D) in APP/PS1+DLT or APP/PS1+AAV‐*si*‐*5HT_2A_R* mice compared with those in APP/PS1 mice.

#### 2.6.2 DLT treatment repressed NLRP3 inflammasome in the hippocampus CA1 region of APP/PS1 mice by antagonizing 5HT_2A_R

In addition, we also inspected the potential of DLT treatment in repressing NLRP3 inflammasome by immunofluorescence assay. As indicated in Figure 4e–g, treatment of DLT or AAV‐*si*‐*5HT_2A_R* obviously decreased NLRP3‐positive puncta number (*F*_3,6_ = 134.9, *p* < 0.0001) and area (*F*_3,9_ = 9.459, *p* = 0.0038) in the hippocampus CA1 region of APP/PS1 mice.

#### 2.6.3 DLT treatment suppressed microglial NF‐κB nuclear translocation in the hippocampus of APP/PS1 mice by antagonizing 5HT_2A_R

Since NF‐κB is a nuclear transcription factor regulating the transcription of inflammatory cytokines and NLRP3 (Zhong et al., 2019), NF‐κB nuclear translocation was detected in microglia of hippocampus CA1 region of APP/PS1 mice. As shown in Figure 4h‐i, DLT or AAV‐*si*‐*5HT_2A_R* treatment inhibited NF‐κB nuclear translocation in microglia (*F*_3,6_ = 45.33, *p* = 0.0002).

#### 2.6.4 DLT treatment promoted M1 to M2 microglial polarization in the brain of APP/PS1 mice by antagonizing 5HT_2A_R

In AD pathology, Aβ promotes classical M1 microglial polarization exacerbating neuroinflammation and neuronal death, and microglial M2 phenotype is involved in anti‐inflammatory process (Paasila et al., 2019).

It was noticed that microglia could not yet be specifically isolated from several CD11b‐positive cell populations by magnetic activated cell sorting due to the high homology between microglia and macrophages/monocytes (Deininger et al., 2000) and microglia and macrophages share the same marker proteins including CD206 (Ji et al., 2018); there is currently no specific M2 polarized marker protein targeting only microglia and CD206 has been applied to label M2‐type microglia. In our assay, CD206 was used to identify M2‐type microglia from separated microglia by CD11b magnetic beads, which might be disturbed by infiltration of peripheral macrophages in the brain but will not affect our conclusion.

Here, we examined the potential of DLT treatment in promoting M1 microglial polarization in the brain of APP/PS1 mice by flow cytometry assay with NOS2‐PE (M1 phenotype) and CD206‐APC (M2 phenotype) antibodies. Microglia were isolated with CD11b magnetic beads for polarization assay (Pluvinage et al., 2019; Ulland et al., 2017; Wang et al., 2018).

As shown in Figure 4j, more NOS2 positive and less CD206 positive microglia were found in the brains of APP/PS1 mice compared with those of WT mice, indicative of M1 polarization in microglia. By contrast, less NOS2 positive and more CD206 positive microglia were determined in the brains of APP/PS1+DLT or APP/PS1 + AAV‐*si*‐*5HT_2A_R* mice in comparison with those of APP/PS1 mice.

Notably, no significant difference was found in the suppression of neuroinflammation (inflammatory factors, NF‐κB nuclear translocation, and M1/M2 microglial polarization) between APP/PS1 + AAV‐*si*‐*5HT_2A_R* + DLT mice and APP/PS1 + AAV‐*si*‐*5HT_2A_R* mice.

Collectively, all results demonstrated that DLT treatment suppressed neuroinflammation in the brains of APP/PS1 mice by antagonizing 5HT_2A_R.

#### 2.6.5 DLT ameliorated AD‐like pathology independent of targeting H1 receptor

Additionally, given that DLT was ever reported to be an antagonist of H1 receptor (H1R) (M. Chen et al., 2015), assays with H1R siRNA were also performed in primary microglia. As indicated in Figure S7A‐G, DLT ameliorated AD‐like pathology (phagocytosis, autophagy and inflammation. See details in Appendix S1) independent of H1R targeting.

### 2.7 DLT treatment regulated autophagy and inflammation in APP/PS1 mice through 5HT_2A_R/cAMP/PKA/CREB/Sirt1 pathway

Sirt1 as a deacetylase plays a potent role in regulating inflammation, neuroprotection and autophagy (Donmez & Outeiro, 2013), and 5HT_2A_R antagonism promotes cAMP accumulation leading to activation of PKA/CREB pathway (Nagatomo et al., 2004). In addition, cAMP‐response element binding protein (CREB) as a transcriptional factor functions potently in mediating Sirt1 transcription and is critical for cognitive improvement (Fusco et al., 2016). With these facts, we investigated the potential mechanisms underlying the regulation of DLT treatment against autophagy and inflammation in the brains of APP/PS1 mice.

#### 2.7.1 DLT treatment regulated autophagy and inflammation through 5HT_2A_R/Sirt1 signaling

**FIGURE 5:**
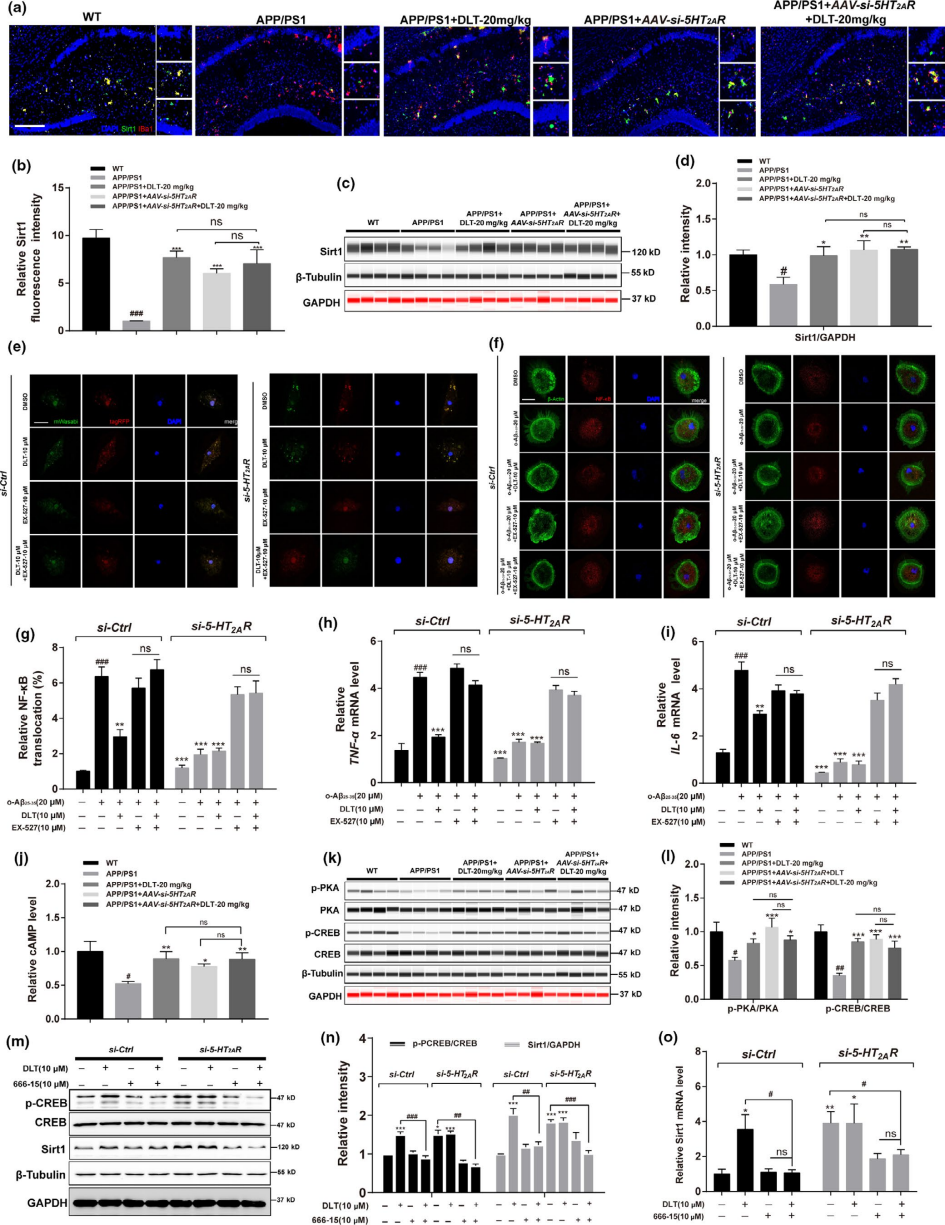
DLT treatment promoted autophagy and suppressed inflammation in APP/PS1 mice through 5‐HT_2A_R/cAMP/PKA/CREB/Sirt1 pathway. (a) Immunofluorescence assay and (b) its quantification results demonstrated that treatment of DLT or AAV‐*si*‐*5HT_2A_R* increased the protein level of Sirt1 in the hippocampus (CA1 region) of APP/PS1 mice (*n* = 4). Scale bar: 100 µm. (c) Jess and (d) its quantification results demonstrated that treatment of DLT or AAV‐*si*‐*5HT_2A_R* increased the protein level of Sirt1 in the brains of APP/PS1 mice (*n* = 4). (e) The results of autophagic flux assay demonstrated that DLT or *si*‐*5HT_2A_R*‐induced autophagy stimulation was abolished by co‐treatment of Sirt1 inhibitor Ex‐527 (*n* = 4). Scale bar: 5 µm. (f) Immunofluorescence assay and (g) its quantification results demonstrated that co‐treatment of Ex‐527 abolished the suppression of NF‐κB nuclear translocation induced by treatment of DLT or *si*‐*5HT_2A_R* in primary microglia (*n* = 4). Scale bar: 5 µm. (h, i) RT‐PCR results demonstrated that co‐treatment of Ex‐527 abolished the suppression of inflammatory cytokines transcription induced by treatment of DLT or *si*‐*5HT_2A_R* in primary microglia (*n* = 3). (j) Treatment of DLT or AAV‐*si*‐*5HT_2A_R* increased cAMP level in the brains of APP/PS1 mice (*n* = 5). (k) Jess assay and (l) its quantification results demonstrated that treatment of DLT or AAV‐*si*‐*5HT_2A_R* increased the level of phosphorylated PKA/CREB in the brains of APP/PS1 mice (*n* = 4). (m) Western blot assay and (n) its quantification results demonstrated that co‐treatment of CREB inhibitor 666–15 abolished the upregulation of Sirt1 protein level induced by treatment of DLT and *si*‐*5HT_2A_R* in BV2 cells (*n* = 3). (o) RT‐PCR results demonstrated that co‐treatment of CREB inhibitor 666–15 abolished the upregulation of Sirt1 mRNA level induced by treatment of DLT and *si*‐*5HT_2A_R* in BV2 cells (*n* = 3). All values were presented as the mean ± SEM. For animal tissue assays, ^#^ *p* < 0.05, ^###^ *p* < 0.001 compared with WT group by *t* test. *p < 0.05 compared with APP/PS1 group by two‐way ANOVA. For cell assays, ^###^ *p* < 0.001 compared with *si*‐*Ctrl*, **p < 0.01, ***p < 0.001 compared with *si*‐*Ctrl*+o‐Aβ_25‐35_ by one‐way ANOVA

To our expect, results of immunofluorescence (Figure 5a,b), Jess assay (Figure 5c,d and Figure S8A) and quantitative RT‐PCR (Figure S8B) assays demonstrated that DLT or AAV‐*si*‐*5HT_2A_R* treatment upregulated the protein (*F*_4,15_ = 4.203, *p* = 0.0201) and mRNA levels (*F*_4,25_ = 5.659, *p* = 0.0022) of Sirt1 in the brains of APP/PS1 mice. Notably, no significant difference was found in Sirt1 regulation within these assays between APP/PS1+AAV‐*si*‐*5HT_2A_R*+DLT mice and APP/PS1+AAV‐*si*‐*5HT_2A_R* mice.

Moreover, Sirt1 inhibitor Ex‐527 (Hubbard & Sinclair, 2014) was applied to inspect whether Sirt1 regulation was required for the DLT‐mediated autophagy stimulation and inflammation suppression in primary microglia. As shown in Figure 5e, co‐treatment of Ex‐527 efficiently blocked the autophagy stimulation induced by DLT or *si*‐*5HT_2A_R* treatment. Additionally, results of immunofluorescence and RT‐PCR assays (Figure 5f–i) demonstrated that co‐treatment of Ex‐527 abolished the activity of DLT or *si*‐*5HT_2A_R* in suppressing NF‐κB nuclear translocation (*F*_9,110_ = 51.65, *p* < 0.0001) and pro‐inflammatory cytokine (TNF‐α and IL‐6) transcription (*F*_9,20_ = 72.55, *p* < 0.0001) (*F*_9,20_ = 59.76, *p* < 0.0001) in primary microglia.

Together, all results indicated that DLT treatment stimulated autophagy and suppressed inflammation in APP/PS1 mice through 5HT_2A_R/Sirt1 signaling.

#### 2.7.2 DLT treatment upregulated Sirt1 by regulating 5HT_2A_R/cAMP/PKA/CREB pathway

Next, Western blot assay was carried out in the brain homogenate of the mice to detect the potential effect of DLT on cAMP/PKA/CREB pathway in APP/PS1 mice. As shown in Figure 5j–l, DLT or AAV‐*si*‐*5HT_2A_R* treatment efficiently upregulated cAMP level (*F*_4,9_ = 7.041, *p* = 0.0098) and p‐PKA/p‐CREB (*F*_3,24_ = 16.06, *p* < 0.0001) in APP/PS1 mice, and there was no significant difference in the regulation of these proteins between APP/PS1 + AAV‐*si*‐*5HT_2A_R*+DLT mice and APP/PS1 + AAV‐*si*‐*5HT_2A_R* mice.

Moreover, CREB inhibitor 666‐15 (Xie et al., 2017) was applied to verify whether CREB signaling was required for the DLT‐mediated Sirt1 upregulation in response to 5HT_2A_R antagonism by Western blot and quantitative RT‐PCR assays in BV2 cells. As shown in Figure 5m–o, treatment of 666‐15 blocked the capability of DLT or *si*‐*5HT_2A_R* treatment in promoting the protein (*F*_15,32_ = 24.83, *p* < 0.0001) and mRNA levels (*F*_7,16_ = 4.854, *p* < 0.0054) of Sirt1.

Taken together, all results demonstrated that DLT treatment promoted autophagy and suppressed inflammation in APP/PS1 mice through 5HT_2A_R/cAMP/PKA/CREB/Sirt1 pathway.

### 2.8 DLT‐mediated 5HT_2A_R/cAMP/PKA/CREB/GR signaling was responsible for microglial phagocytic receptor TLR2/4 regulation

Given that glucocorticoid receptor (GR) as a nuclear transcription factor is activated by CREB and binds to a specific DNA sequence on target genes including TLR2/4 for regulating gene transcription (Novaes et al., 2017) and DLT treatment has been determined to promote microglial phagocytosis by upregulating TLR2/4, we next inspected whether CREB/GR signaling was involved in DLT‐mediated TLR2/4 upregulation.

#### 2.8.1 DLT regulated GR by targeting 5HT_2A_R

**FIGURE 6:**
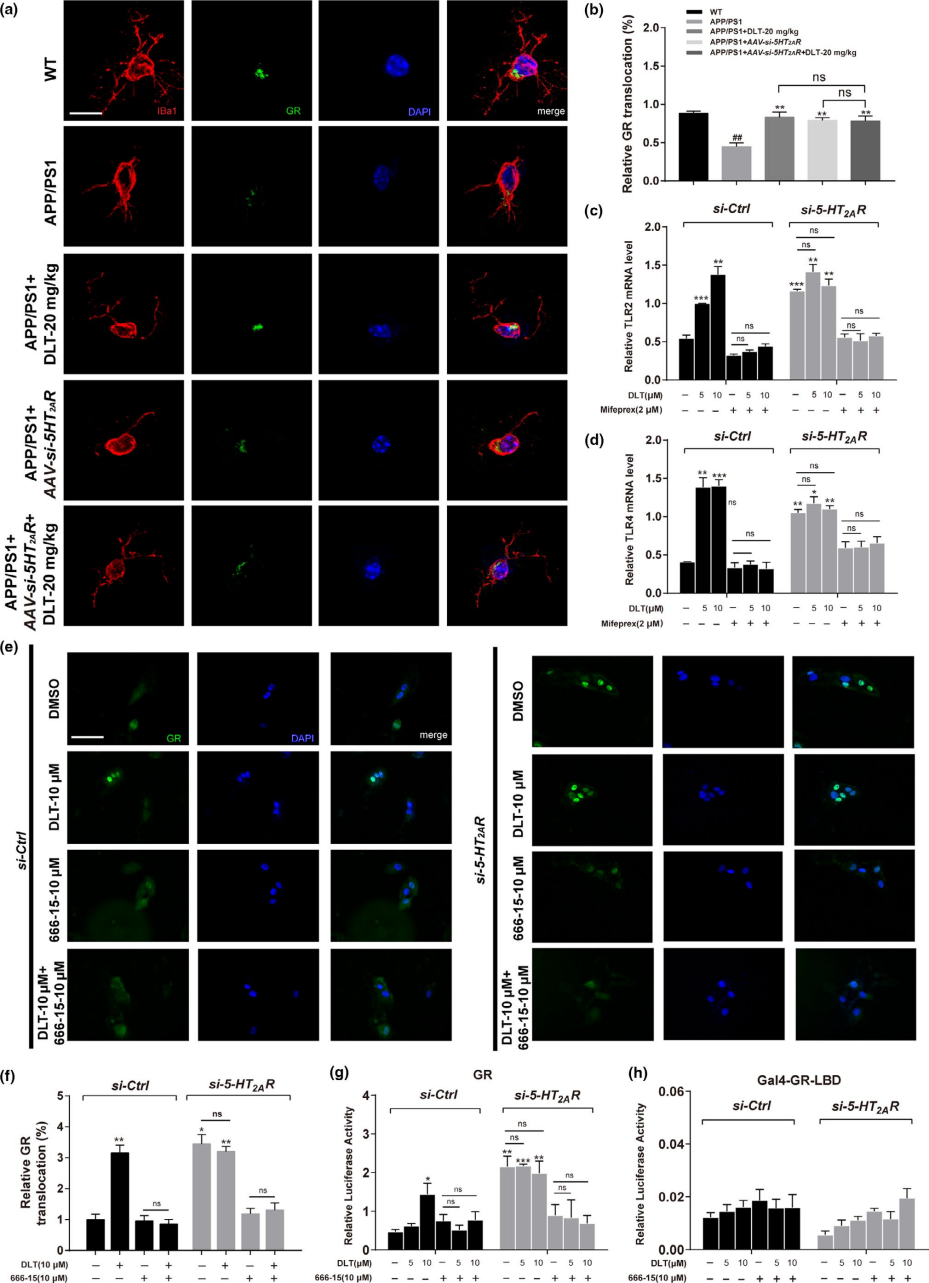
DLT regulated microglial phagocytic receptor TLR2/4 through 5HT_2A_R/cAMP/PKA/CREB/GR signaling. (a) Immunofluorescence assay and (b) its quantification results demonstrated that treatment of DLT or AAV‐*si*‐*5HT_2A_R* stimulated GR nuclear translocation in hippocampal microglia of APP/PS1 mice (brain slice, *n* = 4; cell, *n* = 12). Scale bar: 10 µm. (c, d) RT‐PCR results demonstrated that co‐treatment of GR inhibitor Mifeprex abolished the upregulation of TLR2/4 induced by the treatment of DLT or *si*‐*5HT_2A_R* in BV2 cells (*n* = 3). (e) Immunofluorescence assay and (f) its quantification results demonstrated that co‐treatment of CREB inhibitor 666‐15 abolished the promotion of GR nuclear translocation induced by the treatment of DLT or *si*‐*5HT_2A_R* in GR‐U2OS cells (*n* = 4). Scale bar: 100 µm. (g, h) Results of transactivation and mammalian one‐hybrid assays demonstrated that co‐treatment of 666‐15 abolished the promotion of GR transactivation induced by the treatment of DLT or *si*‐*5HT_2A_R* in HEK‐293 T cells, implying that the DLT‐mediated GR activation was independent of the direct combination of GR with DLT (*n* = 3). All values were presented as the mean ± SEM. For animal tissue assays, ^##^ *p* < 0.001 compared with WT group by *t* test. *p < 0.05, **p < 0.01, ***p < 0.001 compared with APP/PS1 group mice by two‐way ANOVA. For cell assays, *p < 0.05, **p < 0.01, ***p < 0.001 compared with *si*‐*Ctrl* by one‐way ANOVA

Immunofluorescence assay results (Figure 6a,b) indicated that DLT or AAV‐*si*‐*5HT_2A_R* treatment promoted microglial GR nuclear translocation (*F*_3,6_ = 9.655, *p* = 0.0103) in the hippocampus of APP/PS1 mice, and no significant difference was found in the level of GR nuclear translocation between APP/PS1 + AAV‐*si*‐*5HT_2A_R*+DLT mice and APP/PS1 + AAV‐*si*‐*5HT_2A_R* mice. Thus, these results implied that DLT regulated GR by targeting 5HT_2A_R.

#### 2.8.2 DLT upregulated TLR2/4 expression through activating GR

Next, GR antagonist Mifeprex (Xu et al., 2018) was applied in the assay against BV2 cells, and the results (Figure 6c,d) demonstrated that co‐treatment of Mifeprex efficiently blocked the capability of DLT or *si*‐*5HT_2A_R* in upregulating TLR2/4 mRNA level (*F*_11,24_ = 16.26, *p* < 0.0001) (*F*_11,24_ = 10.56, *p* < 0.0001). Thus, all results implied that DLT upregulated TLR2/4 expression through activating GR.

#### 2.8.3 DLT‐mediated CREB activation was required for GR nuclear translocation and transactivation

By considering that DLT treatment has been determined to activate cAMP/PKA/CREB pathway, we next investigated whether DLT‐mediated CREB regulation was required for GR nuclear translocation and transactivation.

In the assay, the GFP‐GR‐U2OS cell line overexpressing GR was at first used for detecting GR nuclear translocation. As shown in Figure 6e,f, DLT or *si*‐*5HT_2A_R* treatment upregulated GR nuclear translocation, but such upregulation was blocked by co‐treatment of CREB inhibitor 666–15 (*F*_7,88_ = 19.56, *p* < 0.0001). Next, GR transactivation activity (*F*_11,24_ = 7.426, *p* = 0.0007) was detected in HEK‐293T cells. As indicated in Figure 6g, DLT or *si*‐*5HT_2A_R* treatment stimulated luciferase gene expression, indicative of the promotion of GR transactivation, but such promotion was blocked by co‐treatment of 666‐15. Additionally, mammalian one‐hybrid assay result (Figure 6h) also indicated that DLT or *si*‐*5HT_2A_R* treatment had no impacts on reporter gene expression (*F*_11,24_ = 1.899, *p* = 0.0404). Thus, this result implied that the DLT‐mediated GR activation was not attributable to the direct binding of GR with DLT.

Together, all results demonstrated that DLT‐mediated 5HT_2A_R/cAMP/PKA/CREB/GR signaling was responsible for microglial phagocytic receptor TLR2/4 regulation.

## 3 DISCUSSION

5HT_2A_R dysfunction links to a series of disorders, including schizophrenia, depression/anxiety, and drug addiction (Zhang & Stackman, 2015). Antagonists of 5HT_2A_R (e.g., Pimavanserin; Clozapine (Joshi et al., 2017)) as antipsychotics have been clinically used to improve symptoms of depression or anxiety of psychiatric patients by regulating depressive release of dopamine (Landolt & Wehrle, 2009). Here, we determined that 5HT_2A_R antagonism efficiently alleviated AD‐like pathology in APP/PS1 mice, and the underlying mechanism has been intensively investigated. Our findings have strongly provided new evidence on the role of 5HT_2A_R antagonism in microglia regulation. Moreover, DLT is currently a clinically antiallergic drug, and its obtained preclinical and clinical data including the efficient amelioration of AD‐like pathology in the current work should no doubt provide valuable references for subsequent development of anti‐AD drug based on this “old drug”.

Given the crucial beneficial role of microglial phagocytosis in synapse regeneration and microenvironment restoration, targeting microglia has been accepted as a promising strategy for drug discovery against AD (Piirainen et al., 2017). TLR2/4 signaling participates in the process of microglial recognition and phagocytosis of neurotoxic Aβ deposits (Vijayraghavan et al., 2018), and upregulations of CD14 and TLR2/4 have been determined in the brains of AD patients (Cameron & Landreth, 2010). TLR2/4 activation promoted cell uptake of AD‐associated amyloid peptide and reduced Aβ level (Fiebich et al., 2018), addressing the potency of TLR2/4 regulation in Aβ clearance. Here, we also determined that DLT elevated microglial phagocytosis through activation of GR/TLR2/4 signaling. Notably, TLRs activation induces inflammation through NF‐κB signaling, as exemplified by the findings that Aβ stimulated pro‐inflammatory factors by promoting TLR2/4 (Song et al., 2018) and curcumin suppressed inflammatory by inhibiting TLR4 (Gao et al., 2019). However, DLT was here determined to inhibit NF‐κB signaling by targeting Sirt1, thus potentially offsetting the abovementioned adverse effects caused by upregulation of TLR2/4.

As a deacetylase, Sirt1 is ubiquitously expressed in brains and deacetylates a number of transcription factors. It functions potently in inflammation, mitochondrial biogenesis, fatty acid oxidation, and mobilization (Zhang et al., 2020). For example, Resveratrol ameliorated mitochondrial dysfunction and cognitive impairment of AD model mice (Lagouge et al., 2006) by activating Sirt1. Here, we determined that DLT upregulated 5HT_2A_R‐mediated Sirt1 expression to repress Aβ level by stimulating autophagy and protect against neuroinflammation by suppressing NF‐κB translocation through 5HT_2A_R/cAMP/PKA/CREB/Sirt1 signaling pathway. cAMP/PKA/CREB signaling is tightly linked to neuronal activities including energy production, metabolic processes, synaptic physiology, neuronal plasticity, and long‐term memory formation (Behravanfar et al., 2017). To our knowledge, our work might be the first report presenting the cross talk between 5HT_2A_R and Sirt1 signaling. All results have thereby highlighted the potential of DLT in the treatment of AD and other related neurodegenerative diseases.

GR as a ligand‐dependent transcription factor is tightly involved in the homeostasis of glucose/lipid and immune response (Hudson et al., 2018). DLT was determined to activate GR through 5HT_2A_R/cAMP/PKA/CREB/GR pathway. Interestingly, a 10‐year large‐scale report revealed that people taking antipsychotics like 5HT_2A_R antagonists easily develop overweight or obesity, although no underlying mechanism was yet disclosed (Wilkins & Sambamoorthi, 2011). Here, it was tentatively proposed that the side effects of overweight or obesity for the users taking 5HT_2A_R antagonists might be largely related to this 5HT_2A_R‐mediated GR regulation.

In conclusion, we determined that DLT effectively improved cognitive impairment in APP/PS1 mice by improving microglial dysfunction. Briefly, DLT reduced amyloid plaque deposition by promoting microglial Aβ phagocytosis and degradation, and ameliorated innate immune response by polarizing microglia to an anti‐inflammatory phenotype. It stimulated autophagy process and repressed neuroinflammation through 5HT_2A_R/cAMP/PKA/CREB/Sirt1 pathway, and activated GR nuclear translocation to upregulate the transcriptions of phagocytic receptors TLR2/4 in response to microglial phagocytosis stimulation. However, it was worth noting that a series of cell‐based assay results also revealed that DLT treatment attenuated Aβ‐induced cell viability repression, cell apoptosis, and caspase 3‐dependent apoptotic pathway activation in primary neurons (Figure S14A–E), indicative of its potential in improving neuronal damage. These results thus suggested that DLT may also perform beneficial impacts on nonmicroglia populations such as neurons. Together, our work has highly supported that 5HT_2A_R antagonism is a promising therapeutic strategy for AD and highlighted the potential of DLT in the treatment of this disease.

## 4 EXPERIMENTAL PROCEDURES

### 4.1 Study design

The goals of the study were to evaluate the potential of 5‐HT_2A_R antagonist Desloratadine in the amelioration of AD‐like pathology of APP/PS1 mice (functional behavior, synaptic integrity and plasticity, Aβ pathology, and neuroinflammation) and investigate the underlying mechanism by assay against APP/PS1 mice with 5‐HT_2A_R knockdown in the brain by injection of AAV‐ePHP‐*si*‐*5*‐*HT_2A_R*. For all animal studies, mice were litter‐matched, age‐matched, and gender‐matched to keep all data agree with each other. Completely random grouping design and exploratory experimental research were performed based on the experimental animals.

Sample sizes were chosen according to the previous experiences with AD related research in our laboratory using the same APP/PS1 model mice. Investigators who conducted the experiments or analyzed the data were blinded to group. Assays of histology and immunostaining of tissue sections, and Western blot, ELISA and RT‐PCR of brain tissues were performed. Cell‐based assays against primary microglia or cell lines were carried out to verify the conclusion of animal experiments.

### 4.2 Statistical analysis

All cell experiments were performed in triplicate to obtain three independently data. Each study was completed with listed number of samples in figure legends. All data were presented as mean ± SEM, and statistical *p* < 0.05 was considered significantly. *t* Test was performed to analyze the significant difference between WT and APP/PS1. Two‐way ANOVA with Fisher's LSD test was performed to analyze the significant differences among APP/PS1, APP/PS1 + DLT, APP/PS1 + AAV‐si‐5HT_2A_R, and APP/PS1 + AAV‐si‐5HT_2A_R + DLT. For cell assay, one‐way ANOVA with Dunnett's post‐test was performed to analyze the significant difference between multiple treatments and the control. The data were analyzed for statistical significance using the graphing program GraphPad Prism 7.

## CONFLICT OF INTEREST

The authors declare that they have no conflict of interest. All institutional and national guidelines for the care and use of laboratory animals were followed.

## AUTHOR CONTRIBUTIONS

X.S. and J.L. designed the study. X.S. reviewed the manuscript. X.W.J and W.Q.L. detected the antagonism ability of DLT on 5‐HT_2A_R. J.L., C.Z.Z., J.L.L., and X.L.Z. performed the animal and cell experiments. J.L. analyzed and interpreted data. J.L. wrote the manuscript. J.L., X.S., J.Y.W., Y.L., and Z.X.T. are the guarantors of this work and, as such, have full access to all data in the study and take responsibility for the integrity of the data and the accuracy of the data analysis. All authors approved the manuscript.

## Supporting information

Supplementary Material

Fig S1

Fig S2

Fig S3

Fig S4

Fig S5

Fig S6

Fig S7

Fig S8

Fig S9

Fig S10

Fig S11

Fig S12

Fig S13

Fig S14

## Data Availability

The data sets used and/or analyzed during the current study are available from the corresponding author on reasonable request.

## References

[acel13286-bib-0001] Behravanfar, N. , Abnous, K. , Razavi, B. M. , & Hosseinzadeh, H. (2017). Effects of Crocin on Spatial Memory Impairment Induced by Hyoscine and Its Effects on BDNF, CREB, and p‐CREB Protein and mRNA Levels in Rat Hippocampus. Jundishapur Journal of Natural Pharmaceutical Products, 12(3 supp), 1–10. 10.5812/jjnpp.64315

[acel13286-bib-0002] Brambilla, D. (2017). Drug discovery, development and delivery in Alzheimer's disease. Pharmaceutical Research, 35(1), 3 10.1007/s11095-017-2329-6 29288429

[acel13286-bib-0003] Cameron, B. , & Landreth, G. E. (2010). Inflammation, microglia, and Alzheimer's disease. Neurobiology of Diseases, 37(3), 503–509. 10.1016/j.nbd.2009.10.006 PMC2823849 19833208

[acel13286-bib-0004] Chen, M. , Xu, S. , Zhou, P. , He, G. , Jie, Q. , & Wu, Y. (2015). Desloratadine citrate disodium injection, a potent histamine H(1) receptor antagonist, inhibits chemokine production in ovalbumin‐induced allergic rhinitis guinea pig model and histamine‐induced human nasal epithelial cells via inhibiting the ERK1/2 and NF‐kappa B signal cascades. European Journal of Pharmacology, 767, 98–107. 10.1016/j.ejphar.2015.10.014 26455479

[acel13286-bib-0005] Chen, Z. , Jalabi, W. , Hu, W. , Park, H.‐J. , Gale, J. T. , Kidd, G. J. , Bernatowicz, R. , Gossman, Z. C. , Chen, J. T. , Dutta, R. , & Trapp, B. D. (2014). Microglial displacement of inhibitory synapses provides neuroprotection in the adult brain. Nature Communications, 5, 4486 10.1038/ncomms5486 PMC4109015 25047355

[acel13286-bib-0006] Currais, A. , Prior, M. , Dargusch, R. , Armando, A. , Ehren, J. , Schubert, D. , & Maher, P. (2014). Modulation of p25 and inflammatory pathways by fisetin maintains cognitive function in Alzheimer's disease transgenic mice. Aging Cell, 13(2), 379–390. 10.1111/acel.12185 24341874 PMC3954948

[acel13286-bib-0007] Deininger, M. H. , Seid, K. , Engel, S. , Meyermann, R. , & Schluesener, H. J. (2000). Allograft inflammatory factor‐1 defines a distinct subset of infiltrating macrophages/microglial cells in rat and human gliomas. Acta Neuropathologica, 100(6), 673–680. 10.1007/s004010000233 11078219

[acel13286-bib-0008] Donmez, G. , & Outeiro, T. F. (2013). SIRT1 and SIRT2: Emerging targets in neurodegeneration. EMBO Molecular Medicine, 5(3), 344–352. 10.1002/emmm.201302451 23417962 PMC3598076

[acel13286-bib-0009] Fiebich, B. L. , Batista, C. R. A. , Saliba, S. W. , Yousif, N. M. , & de Oliveira, A. C. P. (2018). Role of microglia TLRs in neurodegeneration. Frontiers in Cellular Neuroscience, 12, 329 10.3389/fncel.2018.00329 30333729 PMC6176466

[acel13286-bib-0010] Fusco, S. , Leone, L. , Barbati, S. A. , Samengo, D. , Piacentini, R. , Maulucci, G. , Toietta, G. , Spinelli, M. , McBurney, M. , Pani, G. , & Grassi, C. (2016). A CREB‐Sirt1‐Hes1 circuitry mediates neural stem cell response to glucose availability. Cell Reports, 14(5), 1195–1205. 10.1016/j.celrep.2015.12.092 26804914

[acel13286-bib-0011] Gao, Y. Y. , Zhuang, Z. , Lu, Y. , Tao, T. , Zhou, Y. , Liu, G. J. , Wang, H. , Zhang, D. D. , Wu, L. Y. , Dai, H. B. , Li, W. , & Hang, C. H. (2019). Curcumin mitigates neuro‐inflammation by modulating microglia polarization through inhibiting TLR4 axis signaling pathway following experimental subarachnoid hemorrhage. Frontiers in Neuroscience, 13, 1223 10.3389/fnins.2019.01223 31803007 PMC6872970

[acel13286-bib-0012] Hubbard, B. P. , & Sinclair, D. A. (2014). Small molecule SIRT1 activators for the treatment of aging and age‐related diseases. Trends in Pharmacological Sciences, 35(3), 146–154. 10.1016/j.tips.2013.12.004 24439680 PMC3970218

[acel13286-bib-0013] Hudson, W. H. , Vera, I. M. S. , Nwachukwu, J. C. , Weikum, E. R. , Herbst, A. G. , Yang, Q. , & Ortlund, E. A. (2018). Cryptic glucocorticoid receptor‐binding sites pervade genomic NF‐kappaB response elements. Nature Communications, 9(1), 1337 10.1038/s41467-018-03780-1 PMC5889392 29626214

[acel13286-bib-0014] Hung, Y. Y. , Huang, K. W. , Kang, H. Y. , Huang, G. Y. , & Huang, T. L. (2016). Antidepressants normalize elevated Toll‐like receptor profile in major depressive disorder. Psychopharmacology (Berl), 233(9), 1707–1714. 10.1007/s00213-015-4087-7 26415953 PMC4828490

[acel13286-bib-0015] Ji, J. , Xue, T. F. , Guo, X. D. , Yang, J. , Guo, R. B. , Wang, J. , & Sun, X. L. (2018). Antagonizing peroxisome proliferator‐activated receptor gamma facilitates M1‐to‐M2 shift of microglia by enhancing autophagy via the LKB1‐AMPK signaling pathway. Aging Cell, 17(4), e12774 10.1111/acel.12774 29740932 PMC6052482

[acel13286-bib-0016] Joshi, R. S. , Quadros, R. , Drumm, M. , Ain, R. , & Panicker, M. M. (2017). Sedative effect of Clozapine is a function of 5‐HT2A and environmental novelty. European Neuropsychopharmacology, 27(1), 70–81. 10.1016/j.euroneuro.2016.10.007 27955831

[acel13286-bib-0017] Lagouge, M. , Argmann, C. , Gerhart‐Hines, Z. , Meziane, H. , Lerin, C. , Daussin, F. , & Auwerx, J. (2006). Resveratrol improves mitochondrial function and protects against metabolic disease by activating SIRT1 and PGC‐1alpha. Cell, 127(6), 1109–1122. 10.1016/j.cell.2006.11.013 17112576

[acel13286-bib-0018] Landolt, H. P. , & Wehrle, R. (2009). Antagonism of serotonergic 5‐HT2A/2C receptors: mutual improvement of sleep, cognition and mood? European Journal of Neuroscience, 29(9), 1795–1809. 10.1111/j.1460-9568.2009.06718.x 19473234

[acel13286-bib-0019] Lee, C. Y. D. , Daggett, A. , Gu, X. , Jiang, L.‐L. , Langfelder, P. , Li, X. , Wang, N. , Zhao, Y. , Park, C. S. , Cooper, Y. , Ferando, I. , Mody, I. , Coppola, G. , Xu, H. , & Yang, X. W. (2018). Elevated TREM2 gene dosage reprograms microglia responsivity and ameliorates pathological phenotypes in Alzheimer's disease models. Neuron, 97(5), 1032–1048.e1035. 10.1016/j.neuron.2018.02.002 29518357 PMC5927822

[acel13286-bib-0020] Lippold, K. , & Dewey, W. (2017). The role of 5‐HT2a/2c receptors in nociception and opioid antinociception: A review of the preclinical literature. Current Treatment Options in Psychiatry, 4(2), 210–220. 10.1007/s40501-017-0111-3

[acel13286-bib-0021] Moraros, J. , Nwankwo, C. , Patten, S. B. , & Mousseau, D. D. (2017). The association of antidepressant drug usage with cognitive impairment or dementia, including Alzheimer disease: A systematic review and meta‐analysis. Depression and Anxiety, 34(3), 217–226. 10.1002/da.22584 28029715 PMC5347943

[acel13286-bib-0022] Moriguchi, S. , Ishizuka, T. , Yabuki, Y. , Shioda, N. , Sasaki, Y. , Tagashira, H. , Yawo, H. , Yeh, J. Z. , Sakagami, H. , Narahashi, T. , & Fukunaga, K. (2018). Blockade of the KATP channel Kir6.2 by memantine represents a novel mechanism relevant to Alzheimer's disease therapy. Molecular Psychiatry, 23(2), 211–221. 10.1038/mp.2016.187 27777420

[acel13286-bib-0023] Nagatomo, T. , Rashid, M. , Abul Muntasir, H. , & Komiyama, T. (2004). Functions of 5‐HT2A receptor and its antagonists in the cardiovascular system. Pharmacology & Therapeutics, 104(1), 59–81. 10.1016/j.pharmthera.2004.08.005 15500909

[acel13286-bib-0024] Nitsch, R. M. , Deng, M. , Growdon, J. H. , & Wurtman, R. J. (1996). Serotonin 5‐HT2a and 5‐HT2c receptors stimulate amyloid precursor protein ectodomain secretion. Journal of Biological Chemistry, 271(8), 4188–4194. 10.1074/jbc.271.8.4188 8626761

[acel13286-bib-0025] Novaes, L. S. , Dos Santos, N. B. , Batalhote, R. F. P. , Malta, M. B. , Camarini, R. , Scavone, C. , & Munhoz, C. D. (2017). Environmental enrichment protects against stress‐induced anxiety: Role of glucocorticoid receptor, ERK, and CREB signaling in the basolateral amygdala. Neuropharmacology, 113(Pt A), 457–466. 10.1016/j.neuropharm.2016.10.026 27815155

[acel13286-bib-0026] Paasila, P. J. , Davies, D. S. , Kril, J. J. , Goldsbury, C. , & Sutherland, G. T. (2019). The relationship between the morphological subtypes of microglia and Alzheimer's disease neuropathology. Brain Pathology, 29(6), 726–740. 10.1111/bpa.12717 30803086 PMC8028288

[acel13286-bib-0027] Parhizkar, S. , Arzberger, T. , Brendel, M. , Kleinberger, G. , Deussing, M. , Focke, C. , Nuscher, B. , Xiong, M. , Ghasemigharagoz, A. , Katzmarski, N. , Krasemann, S. , Lichtenthaler, S. F. , Müller, S. A. , Colombo, A. , Monasor, L. S. , Tahirovic, S. , Herms, J. , Willem, M. , Pettkus, N. , … Haass, C. (2019). Loss of TREM2 function increases amyloid seeding but reduces plaque‐associated ApoE. Nature Neuroscience, 22(2), 191–204. 10.1038/s41593-018-0296-9 30617257 PMC6417433

[acel13286-bib-0028] Piirainen, S. , Youssef, A. , Song, C. , Kalueff, A. V. , Landreth, G. E. , Malm, T. , & Tian, L. (2017). Psychosocial stress on neuroinflammation and cognitive dysfunctions in Alzheimer's disease: the emerging role for microglia? Neuroscience and Biobehavioral Reviews, 77, 148–164. 10.1016/j.neubiorev.2017.01.046 28185874

[acel13286-bib-0029] Pluvinage, J. V. , Haney, M. S. , Smith, B. A. H. , Sun, J. , Iram, T. , Bonanno, L. , Li, L. , Lee, D. P. , Morgens, D. W. , Yang, A. C. , Shuken, S. R. , Gate, D. , Scott, M. , Khatri, P. , Luo, J. , Bertozzi, C. R. , Bassik, M. C. , & Wyss‐Coray, T. (2019). CD22 blockade restores homeostatic microglial phagocytosis in ageing brains. Nature, 568(7751), 187–192. 10.1038/s41586-019-1088-4 30944478 PMC6574119

[acel13286-bib-0030] Shin, J. Y. , Park, H. J. , Kim, H. N. , Oh, S. H. , Bae, J. S. , Ha, H. J. , & Lee, P. H. (2014). Mesenchymal stem cells enhance autophagy and increase beta‐amyloid clearance in Alzheimer disease models. Autophagy, 10(1), 32–44. 10.4161/auto.26508 24149893 PMC4389879

[acel13286-bib-0031] Song, D. , Jiang, X. , Liu, Y. , Sun, Y. , Cao, S. , & Zhang, Z. (2018). Asiaticoside attenuates cell growth inhibition and apoptosis induced by Abeta1‐42 via inhibiting the TLR4/NF‐kappaB signaling pathway in human brain microvascular endothelial cells. Frontiers in Pharmacology, 9, 28 10.3389/fphar.2018.00028 29441018 PMC5797575

[acel13286-bib-0032] Teixeira, C. M. , Rosen, Z. B. , Suri, D. , Sun, Q. , Hersh, M. , Sargin, D. , Dincheva, I. , Morgan, A. A. , Spivack, S. , Krok, A. C. , Hirschfeld‐Stoler, T. , Lambe, E. K. , Siegelbaum, S. A. , & Ansorge, M. S. (2018). Hippocampal 5‐HT input regulates memory formation and schaffer collateral excitation. Neuron, 98(5), 992–1004.e1004. 10.1016/j.neuron.2018.04.030 29754752 PMC6383566

[acel13286-bib-0033] Ulland, T. K. , Song, W. M. , Huang, S.‐C. , Ulrich, J. D. , Sergushichev, A. , Beatty, W. L. , Loboda, A. A. , Zhou, Y. , Cairns, N. J. , Kambal, A. , Loginicheva, E. , Gilfillan, S. , Cella, M. , Virgin, H. W. , Unanue, E. R. , Wang, Y. , Artyomov, M. N. , Holtzman, D. M. , & Colonna, M. (2017). TREM2 maintains microglial metabolic fitness in Alzheimer's disease. Cell, 170(4), 649–663.e613. 10.1016/j.cell.2017.07.023 28802038 PMC5573224

[acel13286-bib-0034] Vijayraghavan, S. , Major, A. J. , & Everling, S. (2018). Muscarinic M1 receptor overstimulation disrupts working memory activity for rules in primate prefrontal cortex. Neuron, 98(6), 1256–1268.e1254. 10.1016/j.neuron.2018.05.027 29887340

[acel13286-bib-0035] Wang, J. , Xie, L. , Wang, S. , Lin, J. , Liang, J. , & Xu, J. (2018). Azithromycin promotes alternatively activated macrophage phenotype in systematic lupus erythematosus via PI3K/Akt signaling pathway. Cell Death & Disease, 9(11), 1080 10.1038/s41419-018-1097-5 30348950 PMC6197274

[acel13286-bib-0036] Wang, P. , Xu, T. Y. , Wei, K. , Guan, Y. F. , Wang, X. , Xu, H. , & Miao, C. Y. (2014). ARRB1/beta‐arrestin‐1 mediates neuroprotection through coordination of BECN1‐dependent autophagy in cerebral ischemia. Autophagy, 10(9), 1535–1548. 10.4161/auto.29203 24988431 PMC4206533

[acel13286-bib-0037] Wang, X. , Wang, W. , Li, L. , Perry, G. , Lee, H. G. , & Zhu, X. (2014). Oxidative stress and mitochondrial dysfunction in Alzheimer's disease. Biochimica Et Biophysica Acta, 1842(8), 1240–1247. 10.1016/j.bbadis.2013.10.015 24189435 PMC4007397

[acel13286-bib-0038] Wilkins, T. L. , & Sambamoorthi, U. (2011). Antidepressant use, depression, lifestyle factors, and new‐onset diabetes. International Clinical Psychopharmacology, 26(3), 159–168. 10.1097/YIC.0b013e328342ce31 21471774

[acel13286-bib-0039] Xie, F. , Li, B. X. , & Xiao, X. (2017). Design, synthesis and biological evaluation of regioisomers of 666–15 as inhibitors of CREB‐mediated gene transcription. Bioorganic & Medicinal Chemistry Letters, 27(4), 994–998. 10.1016/j.bmcl.2016.12.078 28073675 PMC5296214

[acel13286-bib-0040] Xu, X. , Shi, X. , Chen, Y. , Zhou, T. , Wang, J. , Xu, X. , & Shen, X. (2018). HS218 as an FXR antagonist suppresses gluconeogenesis by inhibiting FXR binding to PGC‐1alpha promoter. Metabolism, 85, 126–138. 10.1016/j.metabol.2018.03.016 29577938

[acel13286-bib-0041] Zhang, G. , & Stackman, R. W. Jr. (2015). The role of serotonin 5‐HT2A receptors in memory and cognition. Frontiers in Pharmacology, 6, 225 10.3389/fphar.2015.00225 26500553 PMC4594018

[acel13286-bib-0042] Zhang, Z. , Shen, Q. , Wu, X. , Zhang, D. , & Xing, D. (2020). Activation of PKA/SIRT1 signaling pathway by photobiomodulation therapy reduces Abeta levels in Alzheimer's disease models. Aging Cell, 19(1), e13054 10.1111/acel.13054 31663252 PMC6974721

[acel13286-bib-0043] Zhong, L. I. , Xu, Y. , Zhuo, R. , Wang, T. , Wang, K. , Huang, R. , Wang, D. , Gao, Y. , Zhu, Y. , Sheng, X. , Chen, K. , Wang, N. A. , Zhu, L. , Can, D. , Marten, Y. , Shinohara, M. , Liu, C.‐C. , Du, D. , Sun, H. , … Chen, X.‐F. (2019). Soluble TREM2 ameliorates pathological phenotypes by modulating microglial functions in an Alzheimer's disease model. Nature Communications, 10(1), 1365 10.1038/s41467-019-09118-9 PMC6433910 30911003

[acel13286-bib-0044] Zhou, C. , Zhong, W. U. , Zhou, J. , Sheng, F. , Fang, Z. , Wei, Y. , Chen, Y. , Deng, X. , Xia, B. , & Lin, J. (2012). Monitoring autophagic flux by an improved tandem fluorescent‐tagged LC3 (mTagRFP‐mWasabi‐LC3) reveals that high‐dose rapamycin impairs autophagic flux in cancer cells. Autophagy, 8(8), 1215–1226. 10.4161/auto.20284 22647982

[acel13286-bib-0045] Zhu, X. , Pan, Y. , Li, Y. , Jiang, Y. , Shang, H. , Gowda, D. C. , Cui, L. , & Cao, Y. (2012). Targeting Toll‐like receptors by chloroquine protects mice from experimental cerebral malaria. International Immunopharmacology, 13(4), 392–397. 10.1016/j.intimp.2012.05.012 22659438

