# Supplementary material for: Antiallergic drug desloratadine as a selective antagonist of 5HT2A receptor ameliorates pathology of Alzheimer's disease model mice by improving microglial dysfunction
Source: Aging Cell. 2020 Dec 24;20(1):e13286. doi: 10.1111/acel.13286 (PMC7811850; doi:10.1111/acel.13286)
Supplement: Supplementary file 1 — Supplementary Material [file ACEL-20-e13286-s001.docx]

Supporting Information

**Experimental procedures**

***Materials***

All cell culture reagents were purchased from Gibco. Desloratadine (DLT), Flopropione, Tropisetron, Phentolamine, Propranolol and Chlorprothixene were purchased from Energy Chemical. Chloroquine (CQ), 3-Methyladenine (3-MA) and Mifepristone (Mifeprex) were obtained from Sigma-Aldrich. EX-527, 666-15, Ketanserin, LY310762, PRX08006, Puerain, Piboserod, SB-742457 and SB-269970 were obtained from MCE. The purity of all compounds used in the experiments is higher than or equal to 98%. *Si-Crtl* and *si-5HT_2A_R* plasmids were purchased from Genomeditech. *Si-Crtl* and *si-H1R* were purchased from GenePharma. AAV-ePHP-*si-5HT_2A_R* was established by GENE.

***Animals***

All animal experiments were performed according to the institutional ethical guidelines on animal care of Nanjing University of Chinese Medicine and maintained under standard conditions at room temperature (22 °C) by a 12 h light/dark cycle.

To rule out the possibility of the development of epilepsy in APP/PS1 mice with C57BL/6 background (Minkeviciene et al., 2009), APP/PS1 mice with B6C3F1 background were thus chosen in our experiments (X. Guo et al., 2018).

Male wild type (WT, B6C3F1) and APP/PS1 (APPswe, PS1dE9, B6C3F1) transgenic mice were originally obtained from Nanjing Institute of Biomedicine (Nanjing, China). The APP/PS1 mice with selective 5HT_2A_R knockdown in the brain was obtained by injecting adeno-associated virus (AAV)-ePHP-*si-5HT_2A_R* (AAV-*si-5HT_2A_R*) through tail vein against APP/PS1 mice at the age of six and a half months (Figure 1B). The effective sequences of *si-RNA* targeting 5HT_2A_R were screened by RT-PCR and western blot (Figure S2A-D) in microglia and BV2 cells and built into AAV-ePHP virus. The knockdown selectivity of AAV-*si-5HT_2A_R* against 5HT_2A_R in the brain over other tissues of APP/PS1 mice was assessed after half a month. Western blot results indicated that intravenous injection of AAV-*si-5HT_2A_R* selectively reduced the expression of 5HT_2A_R in the brain compared with other tissues of APP/PS1 mice (Figure S2E-F).

In the current study, the experimental animals were totally set to five groups. WT male mice (eight mice) in the first group (WT mice) served as negative control and treated with vehicle (6% Tween-80). Thirty-two APP/PS1 male mice were randomly divided into four groups with eight in each group. The APP/PS1 mice in the second group (APP/PS1 mice) were treated with vehicle, in the third group (APP/PS1+DLT mice) were treated with DLT (20 mg/kg/day), in the fourth group (APP/PS1+AAV-*si-5HT_2A_R* mice) were injected with AAV-ePHP-*si-5HT_2A_R* (AAV-*si-5HT_2A_R*) through tail vein and treated with vehicle, and in the fifth group (APP/PS1+AAV-*si-5HT_2A_R*+DLT mice) were injected with AAV-*si-5HT_2A_R* through tail vein and treated with DLT (20 mg/kg/day).

DLT was dissolved in 6% Tween-80, and APP/PS1 mice were treated with DLT by oral gavage administration at the age of seven months considering the good oral availability and blood-brain barrier (BBB) permeability of DLT (Katta et al., 2007).

***Behavioral test***

*Y-maze test-*The test was performed according to the published approach (Souchet et al., 2019) in a maze consisting of three transparent plastic arms (46 × 11 × 25 cm^3^ each), set at a 120° angle relative to each other. During the first trial, an arm (novel arm) was blocked by an opaque door and the mice could freely explore other two arms (familiar arms) for 5 min. Mice were returned to stay their home cages for 5 min, and then returned to the maze and allowed to explore all three arms for 5 min. The maze was carefully cleaned with a 70% ethanol solution between each exploration phase to remove any olfactory cues. All data were collected for animal performance analysis.

*New object recognition test*-The test was performed according to the published approach (Robin et al., 2018) in an open-field apparatus (32 × 32 × 20 cm^3^). The task consisted of 4 sequential daily trials. During the habituation trial (day 1 and 2), mice were placed in the center of the open-field apparatus and allowed to freely explore the space in the absence of any objects for 15min. During the acquisition trial (day 3), mice were placed in the open-field apparatus again in the presence of two identical objects positioned near the two corners and allowed to freely explore space. Finally, the memory test was performed 24 h later (day 4): one of the familiar objects was replaced by a novel object with difference in its shape, color, and texture and mice being left to explore both objects. The apparatus and objects were cleaned with ethanol (70%) before use and between each animal test. All data were collected for animal performance analysis by the discrimination index (DI) that is calculated as the difference between the time spent exploring the novel (TN) and the familiar object (TF) divided by the total exploration time (TN+TF): DI = [TN-TF]/[TN+TF].

*Open-field test*-The test was carried out according to the published approach (Q. Zhong et al., 2019) in an open-field apparatus (32 × 32 × 20 cm^3^). In brief, each mouse was placed in the corner of the open-field apparatus and allowed to freely explore the arena for 5 min. The apparatus was cleaned with ethanol (70%) before use and between each animal testing. All data were collected for animal performance analysis.

*Morris water maze-*The assay was performed according to the published approach (X. D. Guo et al., 2016; Souchet et al., 2019). Briefly, training trials was continuously performed for 12 days and trained 3 times a day. During training trials, the invisible submerged platform was placed in the circular pool (120 cm in diameter, 50 cm deep) filled with milk, the mice were given 90s to find the platform and allowed to stay at the platform for 10s. If the mice failed to find the platform within 90s, the mice were gently pulled from the water onto the platform and kept there for 10s. On the last day, a probe trial was carried out and the platform was removed. The mice were allowed to swim for 90s in search of it. All data were collected for animal performance analysis. For data analysis, the pool was artificially divided into four equal quadrants formed by imaging lines, intersected in the center of the pool at right angles by north, south, east and west.

*Forced-swim test*-The test was performed according to the published approach (Teixeira et al., 2018) in a 1-liter glass beaker (Height: 158 mm; Width: 108 mm) containing water at a temperature of approximately 25ºC. Briefly, each mouse was individually forced to swim in the glass beaker for 6 min. After each trial, the water was changed. All data were collected for animal performance analysis.

***Cell culture***

BV2, GFP-GR-U2OS and HEK-293T cells were grown in DMEM (Gibco) supple­mented with 10% FBS (Gibco) and 100 unit/mL penicillin-streptomycin (Gibco).

Primary microglia were separated from P0 mice brains (within 24 hours of birth). The brain tissues were minced into small pieces, digested with 0.25% trypsin (Gibco) and 200 U/mL DNase (Sigma-Aldrich), and incu­bated for 10 min at room temperature, while digestion was stopped by adding 4 ml DMEM with 10% FBS. Cell fluid was diluted at a density of 600,000 cells/mL on poly-*D*-lysine (Sigma-Aldrich)-coated cell culture flasks. After 7 days, the microglia were dissociated by shaking the flasks several times, harvested by centrifugation (300 g× 10 min), followed by dilution at a density of 50,000 cells/mL on poly-*D*-lysine coated cell culture plates. Cultures were kept in a humidified incubator with 5% CO_2_ at 37 °C.

Primary neuronal cells were separated from embryonic mouse brains (embryonic d 16 to 18). The brain tissues were minced into small pieces, digested with D-Hanks buffer containing 0.125% trypsin (Gibco) and 200 U/mL DNase (Sigma-Aldrich), and incu­bated for 15 min at 37 °C, while digestion was stopped by adding 4 ml DMEM (Gibco) MEM with 10% FBS. Cell fluid was diluted at a density of 600,000 cells/mL on poly-*D*-lysine (Sigma-Aldrich)-coated cell culture plates. After 6 h, the medium was replaced by a Neurobasal medium (Gibco) supplemented with 2% B27, 0.5 μM *L*-glutamine and 50 U/μL penicillin-streptomycin. Cultures were kept in a humidified incubator with 5% CO_2_ at 37 °C.

***Calcium flux assay***

The CHO-K1 cell line stably expressing human Gα16 (CHO-Gα16) was maintained in DMEM/F12 medium (Gibco) containing 10% FBS (Gibco) at 37 °C in a humidified incubator with 5% CO_2_. The cells were transfected into 3μg pcDNA3.1-5HT_2A_R plasmid and seeded into 96-well black/clear tissue culture plates (25,000 cells/well). Primary neurons and microglia were seeded into 96-well black/clear tissue culture plates (20,000 cells/well), and treated with *si-Crtl* or *si-5HT_2A_R*. For calcium flux assay, the cells were loaded with 100 μL Calcium-5 assay kit (Molecular Devices) dissolved in HBSS buffer (Gibco) containing 20 mM HEPES, incubated at 37 °C for 45 min and room temperature for 15 min. 25 μL DLT (20 mM) was then injected into the plate by Flexstation III instrument (Molecular Devices), and intracellular calcium mobilization was continuously detected at an excitation wavelength of 485 nm and emission wavelength of 525 nm for 120 s.

For antagonist related assay, the cells were pre-treated with compounds for 15 min, and 50 nM 5HT was added into the plate before detecting intracellular calcium mobilization. EC_50_ or IC_50_ values were calculated with GraphPad Prism 5 software (GraphPad).

Agonistic activities were expressed as: Response % = [(RFU-RFUmin)/(RFUmax-RFUmin)] ×100%, ((RFU) in compounds-treated cells, (RFUmin) in vehicle-treated cells, and (RFUmax) in 10 μM 5-Hydroxytryptamine (5HT, Sigma-Aldrich) treated cells.

***Electrophysiology test***

Mice were anesthetized with 10% chloral hydrate (Sigma-Aldrich) and decapitated. Brains were rapidly removed from skull and placed in chilled (0-3°C) artificial cerebrospinal fluid (ACSF) containing 126 mM NaCl, 2.5 mM KCl, 1.25 mM NaH_2_PO_4_, 2 mM MgSO_4_, 2mM CaCl_2_, 26 mM NaHCO_3_ and 10 mM glucose. Transverse brain slices were (370 μm thick) prepared by using a vibratome (Leica) and incubated for 0.5 h in continuously oxygenated (95% O_2_, 5% CO_2_) ACSF at 37 °C and then incubated for 1h at room temperature. After a 1.5 h recovery period, a slice was transferred to a submersion-type recording chamber (Molecular Devices) and submerged in ACSF continuously perfused and equilibrated with 95% O_2_ and 5% CO_2_.

In the electrically induced long-term potentiation (LTP) assay, the baseline in resting state was recorded for 10 min, and the LTP in the DG area was then induced by four trains of 100-Hz stimuli with the same intensity of the test stimulus. The strength of synaptic transmission was determined by measuring the initial (20-80% rising phase) slope of field excitatory postsynaptic potentials (fEPSPs). Each brain slice was recorded every 40s for 70 min (10 min baseline and 60 min LTP) and four brain slices from two animals were recorded and used to calculate the means.

***Golgi-Cox staining***

Golgi-Cox staining and spindle number measurement were performed by using the FD Rapid GolgiStain Kit (FD NeuroTechnologies) according to the manufacturer’s protocol. Briefly, mice were anesthetized with 10% chloral hydrate and decapitated. Brains were rapidly removed from skull and placed in 4% paraformaldehyde for 4h. Then, the brains were placed in 50% solution A and 50% solution B for 2 weeks and subsequently transferred to solution C at room temperature in the darkness for 3 days. Then, the tissues were embedded in OCT (SAKURA) and cut into 100 μm-thick sections by using a vibratome (Leica). The sections were mounted on gelatin-coated slides with solution C. After drying at room temperature, the sections were washed with ddH_2_O 5 times and immerged in a mixture containing 25% solution D, 25% solution E and 50% ddH_2_O water for 10 minutes. After washing with ddH_2_O twice, the sections were dehydrated in graded alcohol solutions (70%, 80%, 90% and 100%), cleaned in xylene and covered lipped. Images for neurons were obtained with an automated upright microscope (Leica).

For dendritic spine assay, neurons in CA1 area of hippocampus from different animals were selected for analysis. 10 neurons per brain were analyzed and used for calculation of the mean. The dendritic spines (spinous protrusions on dendritic branches, including stubby, thin and bifurcated) were identified and analyzed by using image J with a NeuroJ plugin according to application guideline (<http://imagescience.org/meijering/software/neuronj/>).

***Immunohistochemistry***

In immunohistochemistry assay against the brain tissues, the right hemisphere was immersed in 4 % paraformaldehyde overnight, and kept in 30% sucrose solution for 14 days until tissues settled down. The tissues were embedded in OCT and cut into 20 μm-thick sections by using a cryomicrotome (Leica).

For detecting the senile plaque in the brain tissues, the Thioflavin S staining (Byotime) was performed according to the published approach.

For detecting Aβ plaque level (endogenous level of total Aβ peptide including several isoforms of Aβ, such as Aβ_37_, Aβ_38_, Aβ_39_, Aβ_40_ and Aβ_42_), NeuN, GFAP, IBa1, TLR2, TLR4, LC3, Sirt1, NF-κB and NLRP3, the slides were incubated in PBST (5% Triton X-100, [Beyotime](http://www.baidu.com/link?url=hNLLFqEdSpatBriugzsEaz0B_p_Kdd0lg6XMU-rPuRFlovVGh3BC9MVIokUzWew7)) for 15min and in 5% BSA for 1h at room temperature, followed by incubation overnight at 4 °C with the primary antibody (dilu­tion 1:400). Then, the slides were washed with PBS by three times and incubated with fluorescent secondary detection antibodies (dilu­tion 1:250). The slides were observed by using a microscope (Leica) and the images were analyzed by the Image J software.

All antibodies have been listed with the specific catalog number and vendor (Table 1).

***Autophagic*** ***flux assay***

Autophagy process was investigated by mTagRFP-mWassbi-LC3 translocation assay (Zhou et al., 2012). Briefly, microglial cells were transfected with mTagRFP-mWassbi-LC3 plasmid via Lipofectamine 2000 (Invitrogen) according to the manufacturer’s protocol. The cells were treated without or with 10 µM DLT for 4 h and then fixed with 4% paraformaldehyde. In the assay, at early stage of autophagosomes biogenesis, puncta of mTagRFP-mWasabi-LC3 accumulated and displayed both green and red fluorescent signals (mRFP+GFP+yellow). Notably, when autophagosomes were fused with lysosomes, they formed acidic autolysosomes (mRFP-GFP+red), and co-located mTagRFP-mWassbi-LC3 emitted only a red signal because the green signal quenched immediately under acidic conditions. The images were acquired by using a Leica DMI8 microscope and analyzed by the Image J software.

***siRNA plasmid*** ***transfection***

For *siRNA* plasmid transfection, BV2 or primary microglial cells were seeded at a density of 50,000 cells/mL on poly-*D*-lysine (Sigma-Aldrich)-coated 24 cell culture plates. After 24 h, the cells were transfected with the plasmid expressing *si-Crtl*, *si-5HT_2A_R or si-H1R* using Lipofectin 2,000 transfection reagent. 48 h later, 5HT_2A_R or H1R knockdown efficiency by *siRNA* was detected by western blot or RT-PCR assay. The protein level of 5HT_2A_R was reduced by about 50% after siRNA transfection in the cells, and the protein level of H1R was reduced by about 60% after siRNA transfection *(si-H1R-3)* in primary microglia.

***Live cell imaging assay***

Phagocytosis of microglia against o-Aβ_42_ was detected by live cell imaging assay. For preparing fluorescent FAM-oligomer Aβ_42_ (FAM-o-Aβ_42_), FAM-labeled-Aβ_42_ was configured as a solution at a concentration of 2 μg/mL according to the manufacturer’s protocol and placed in a cell culture incubator for 7 days. Microglia were seeded overnight in 96-well plates at a density of 100,000 cell/well in 100 μL medium. Then，the cells were transfected with *si*-5HT_2A_R plasmid, and treated with or without DLT or CQ / 3-MA, or with DLT plus CQ / 3-MA, followed by incubation with fluorescent FAM-o-Aβ42 for 4h / 20h (L. Zhong et al., 2019). The phagocytosis or degradation process of microglia against o-Aβ_42_ was recorded by detecting intracellular green fluorescence (IncuCyte). For live cell imaging assay, real-time detection of live cell images was recorded and quantified (level of Aβ phagocytosis / degradation within detection time).

***Western blot and Jess assay***

For cell samples, BV2 cells or microglia were lysed with RIPA buffer (Beyotime) containing protease inhibitor and phos­phatase inhibitor cocktails (Thermo Scientific) on ice for 20 min.

For tissue samples, brain tissues were homogenized with RIPA buffer containing protease inhibitor and phos­phatase inhibitor cocktails and the homogenates were kept on ice for 30min. After centrifuging at 12,000*g* for 15 min at 4 °C, the supernatants were collected, and protein concentration was determined by using BCA protein assay kit (Beyotime). Proteins were mixed with 4×loading buffer (Thermo Scientific) and boiled for 15 min at 95 °C.

Cell or tissue extracts were separated by SDS-PAGE and trans­ferred to a nitrocellulose filter membrane (GE Healthcare). After blocking for 2 h at 37°C, the membranes were incubated with the corresponding antibodies (dilu­tion 1:1000) overnight at 4 °C. The membranes were washed and incubated for 2 h at room temperature with secondary antibodies (dilu­tion 1:3,000) conjugated with horseradish peroxidase (Jackson). The blots were developed visualized by using the Dura detection system (Thermo Scientific).

Jess assay was performed to detect the protein levels in brain tissue samples by using the Jess/Wes Separation 12-230 KDa 8×25 Capillary Cartridges (ProteinSimple, USA) according to the manufacturer’s protocol (Ising et al., 2019). Bands were quantified according to manufacturer’s protocol of antibodies, for APP protein detection, all bands between 100 and 140 kDa were quantified.

All antibodies have been listed with specific catalog number and vendor (Table 1).

| Antibodies | Source | Identifier | Specificities |
| --- | --- | --- | --- |
| SR-2A antibody (A-4) | Santa Cruz | sc-166775 | H, M, R |
| TLR2 Mouse mAb | Santa Cruz | sc-21759 | H, M, R |
| β-Tubulin (9F3) Rabbit mAb | [Cell Signaling Technology](http://www.baidu.com/link?url=gVpHwHg8_YRAjWxpLG8I0cTL7ADMfZP409UdNVZ6Gx_e6EC2GMMX95sD8OGrYUEF&wd=&eqid=e184c2a900016704000000055eb90235) | 2128 | H, M, R |
| Phospho-CREB (Ser133) (87G3) Rabbit mAb | [Cell Signaling Technology](http://www.baidu.com/link?url=gVpHwHg8_YRAjWxpLG8I0cTL7ADMfZP409UdNVZ6Gx_e6EC2GMMX95sD8OGrYUEF&wd=&eqid=e184c2a900016704000000055eb90235) | 9198 | H, M, R |
| [CREB (48H2) Rabbit mAb](https://www.cst-c.com.cn/products/primary-antibodies/creb-48h2-rabbit-mab/9197?site-search-type=Products) | [Cell Signaling Technology](http://www.baidu.com/link?url=gVpHwHg8_YRAjWxpLG8I0cTL7ADMfZP409UdNVZ6Gx_e6EC2GMMX95sD8OGrYUEF&wd=&eqid=e184c2a900016704000000055eb90235) | 9197 | H, M, R |
| Phospho-PKA C (Thr197) Antibody | [Cell Signaling Technology](http://www.baidu.com/link?url=gVpHwHg8_YRAjWxpLG8I0cTL7ADMfZP409UdNVZ6Gx_e6EC2GMMX95sD8OGrYUEF&wd=&eqid=e184c2a900016704000000055eb90235) | 4781 | H, M, R |
| PKA C-α Antibody | [Cell Signaling Technology](http://www.baidu.com/link?url=gVpHwHg8_YRAjWxpLG8I0cTL7ADMfZP409UdNVZ6Gx_e6EC2GMMX95sD8OGrYUEF&wd=&eqid=e184c2a900016704000000055eb90235) | 4782 | H, M, R |
| NeuN (D4G4O) XP^®^ Rabbit mAb | [Cell Signaling Technology](http://www.baidu.com/link?url=gVpHwHg8_YRAjWxpLG8I0cTL7ADMfZP409UdNVZ6Gx_e6EC2GMMX95sD8OGrYUEF&wd=&eqid=e184c2a900016704000000055eb90235) | 24307 | H, M, R |
| IBa1 mAb | Wako | 019-19741 | H, M, R |
| GFAP (E4L7M) XP^®^ Rabbit mAb | [Cell Signaling Technology](http://www.baidu.com/link?url=gVpHwHg8_YRAjWxpLG8I0cTL7ADMfZP409UdNVZ6Gx_e6EC2GMMX95sD8OGrYUEF&wd=&eqid=e184c2a900016704000000055eb90235) | 80788 | H, M, R |
| [Anti-SIRT1 antibody [EPR18239]](https://www.abcam.cn/sirt1-antibody-epr18239-ab189494.html) | Abacm | [ab110304](https://www.abcam.cn/sirt1-antibody-19a7ab4-ab110304.html) | H, M, R |
| TLR4 Antibody | Proteintech | 19811-1-AP | H, M, R |
| β-Amyloid (D3D2N) Mouse mAb | [Cell Signaling Technology](http://www.baidu.com/link?url=gVpHwHg8_YRAjWxpLG8I0cTL7ADMfZP409UdNVZ6Gx_e6EC2GMMX95sD8OGrYUEF&wd=&eqid=e184c2a900016704000000055eb90235) | 15126 | H |
| Anti-BACE1 (AB1) antibody | Sigma | SAB2900206 | H, M, R |
| LC3A/B Antibody | [Cell Signaling Technology](http://www.baidu.com/link?url=gVpHwHg8_YRAjWxpLG8I0cTL7ADMfZP409UdNVZ6Gx_e6EC2GMMX95sD8OGrYUEF&wd=&eqid=e184c2a900016704000000055eb90235) | 4108 | H, M, R |
| [NF-κB p65 (D14E12) XP^®^ Rabbit mAb](https://www.cst-c.com.cn/products/primary-antibodies/nf-kb-p65-d14e12-xp-rabbit-mab/8242?site-search-type=Products) | [Cell Signaling Technology](http://www.baidu.com/link?url=gVpHwHg8_YRAjWxpLG8I0cTL7ADMfZP409UdNVZ6Gx_e6EC2GMMX95sD8OGrYUEF&wd=&eqid=e184c2a900016704000000055eb90235) | [8242](https://www.cst-c.com.cn/products/primary-antibodies/nf-kb-p65-d14e12-xp-rabbit-mab/8242?site-search-type=Products) | H, M, R |
| [VAMP2 (D6O1A) Rabbit mAb](https://www.cst-c.com.cn/products/primary-antibodies/vamp2-d6o1a-rabbit-mab/13508?site-search-type=Products) | [Cell Signaling Technology](http://www.baidu.com/link?url=gVpHwHg8_YRAjWxpLG8I0cTL7ADMfZP409UdNVZ6Gx_e6EC2GMMX95sD8OGrYUEF&wd=&eqid=e184c2a900016704000000055eb90235) | [13508](https://www.cst-c.com.cn/products/primary-antibodies/vamp2-d6o1a-rabbit-mab/13508?site-search-type=Products) | H, M, R |
| Synaptophysin (D35E4) Rabbit mAb | [Cell Signaling Technology](http://www.baidu.com/link?url=gVpHwHg8_YRAjWxpLG8I0cTL7ADMfZP409UdNVZ6Gx_e6EC2GMMX95sD8OGrYUEF&wd=&eqid=e184c2a900016704000000055eb90235) | 5461 | H, M, R |
| [PSD95 (D27E11) XP^®^ Rabbit mAb](https://www.cst-c.com.cn/products/primary-antibodies/psd95-d27e11-xp-rabbit-mab/3450?site-search-type=Products) | [Cell Signaling Technology](http://www.baidu.com/link?url=gVpHwHg8_YRAjWxpLG8I0cTL7ADMfZP409UdNVZ6Gx_e6EC2GMMX95sD8OGrYUEF&wd=&eqid=e184c2a900016704000000055eb90235) | [3450](https://www.cst-c.com.cn/products/primary-antibodies/psd95-d27e11-xp-rabbit-mab/3450?site-search-type=Products) | H, M, R |
| sAPPβ-sw antibody | IBL | 10321 | H |
| [NLRP3 Monoclonal Antibody (768319)](https://www.thermofisher.com/antibody/product/NLRP3-Antibody-Monoclonal/MA5-23919) | Invitrogen | MA5-23919 | H, M |
| [GAPDH Mouse Monoclonal antibody](http://www.ptgcn.com/products/GAPDH-Antibody-60004-1-Ig.htm) | Proteintech | [60004-1](http://www.ptgcn.com/products/GAPDH-Antibody-60004-1-Ig.htm) | H, M, R |
| [Phospho-APP (Thr668) (D90B8) Rabbit mAb](https://www.cst-c.com.cn/products/primary-antibodies/phospho-app-thr668-d90b8-rabbit-mab/6986?site-search-type=Products) | [Cell Signaling Technology](http://www.baidu.com/link?url=gVpHwHg8_YRAjWxpLG8I0cTL7ADMfZP409UdNVZ6Gx_e6EC2GMMX95sD8OGrYUEF&wd=&eqid=e184c2a900016704000000055eb90235) | [6986](https://www.cst-c.com.cn/products/primary-antibodies/phospho-app-thr668-d90b8-rabbit-mab/6986?site-search-type=Products) | H, M, R |
| H1R antibody | Biolab | K17564 | H, M, R |
| APC anti-mouse CD206 | Miltenyi | 141708 | M |
| iNOS Monoclonal Antibody (CXNFT), PE, eBioscience™ | Invitrogen; | 12-5920-82 | M |
| Species Cross-Reactivity Key: **H**-Human, **M**-Mouse, **R**-Rat | | | |

**Table 1. Primary antibodies list.**

***ELISA assay***

Levels of IL-6, TNF-α, IL-4 and IL-10 of the brain tissue were examined according to the protocol of ELISA kit (Jiancheng). In the assay, the brain tissues were homogenized with RIPA buffer containing a protease inhibitor cocktail and the homogenates were kept on ice for 30 min, followed by centrifugation at 20,000g for 30 min at 4 °C. The supernatants were collected and protein concentration was determined by using a BCA protein assay kit (Beyotime).

***Real-time quantitative PCR analysis***

Total mRNA was extracted from cells or brain tissues of mice according to the manufacturer's protocols by using TRIzol reagent (TaKaRa Biotechnology Co). cDNA synthesis was performed with oligo-dT as the primer using reverse-PCR kit (TaKaRa Biotechnology Co). RT-PCR was carried out by using SYBR green PCR core reagent kit (TaKaRa Biotechnology Co).

Primer sequences for analyzing mRNA levels of 5HT_2A_R, TLR2, TLR4, IL-6, TNF-α, Sirt1, IL-4, IL-10 and GAPDH were as follows (Table 2).

| Primers | Forward | Reverse |
| --- | --- | --- |
| mice 5HT_2A_R | CAGGCAAGTCACAGGATAGC | TTAAGCAGAAAGAAAATCCCACAG |
| mice TLR2 | GAG CTC GTA GCA TCC TCT | GCT CTA TGA CTC CCA G |
| mice TLR4 | GAG ATG AAT ACC TCC TTA GTG TTG G | ATT CAA AGA TAC ACC AAC GGC TCT GA |
| mice IL-6 | TAC CAC TCC CAA CAG ACC TG | GGT ACT CCA GAA ACC AGA GG |
| mice TNF-α | CAC CAT GAG CAC AGA AAG CA | TAG ACA GAA GAG CGT GGT GG |
| mice Sirt1 | TAGCCTTGTCAGATAAGGAAGGA | ACAGCTTCACAGTCAACTTTGT |
| mice IL-4 | GGTCTCAACCCCCAGCTAGT | GCCGATGATCTCTCTCAAGTGAT |
| mice IL-10 | GCTCTTACTGACTGGCATGAG | CGCAGCTCTAGGAGCATGTG |
| mice GAPDH | AGAAGGCTGGGGCTCATTTG | AGGGGCCATCCACAGTCTTC |

**Table 2. Primer sequence list.**

***Flow cytometry***

Microglial M1/M2 polarization typing was assessed by flow cytometry according to the manufacturer’s protocol (Miltenyi Biotec). Briefly, single cell suspensions of hippocampus and cortex were obtained. Then, the Percoll solution (GE) was used to remove myelin through gradient density centrifugation. Microglia in cell suspensions were isolated by CD11b magnetic bead sorting. Finally, the microglia were stained with CD206-APC and NOS2-PE antibodies and examined with IntelliCyt's iQue Screener Plus.

Apoptosis assay was performed by using Annexin V-FITC/PI apoptosis detection kit (KeyGen) according to the manufacturer’s protocol. Primary neurons were seeded overnight in 12-well plates at a density of 5×10^5^ cells/well in 1mL medium. Then, primary neurons were co-incubated with Aβ (20 μM) and DLT for 24h and collected. The proportion of cell apoptosis was examined with IntelliCyt's iQue Screener Plus.

***Intracellular cAMP assay***

cAMP level in brain tissues was measured by using cAMP Enzyme Immunoassay Kit (Sigma). The assay details were as described in the manufacturer’s protocols. Tissue samples were rapidly frozen in liquid nitrogen and ground to a fine fragment. Tissue fragments were homogenized in 200 μL of 0.1 M HCl and then centrifuged at 600×g for 10 minutes. The supernatants were extracted, and samples were assayed directly with kit.

***GR nuclear translocation assay***

GFP-GR-U2OS cells were used to detect the nuclear translocation of GR. Briefly, GFP-GR-U2OS cells were seeded at a density of 10,000 cell/well in 96-well plates and cultured overnight. After incubating with the relevant compounds for 6 h, the nuclear translocation of GR was detected by detecting intracellular green fluorescence (Leica).

***Luciferase reporter assay***

Transactivation and mammalian one-hybrid assays were carried out according to the published approach (Xu et al., 2018). Briefly, HEK-293T cells were seeded at a density of 50,000 cell/well in 48-well plates and cultured overnight. Transient transfections (200 ng/well pCI-nGFP-C656G, 200 ng/well pGL3-GRE-Luc and 50 ng/well pRL-SV40 for transactivation assay; 200 ng/well pCMX-Gal4-DBD-GR-LBD, 200 ng/well pUAS-TK-luc and 20 ng/well pRL-SV40 for mammalian one-hybrid assay) were then conducted by Calcium Phosphate Cell Transfection kit (Beyotime). After 6 h, HEK293T cells were incubated with the relevant compounds for 24 h. Finally, firefly and renilla luciferase activities were measured by dual-luciferase reporter assay system kit (Promega).

***Blood-brain barrier permeability assay***

DLT level in the brain or plasma of mice was detected by using a Thermo TSQ Vantage tandem mass spectrometer coupled with an HPLC model U3000 apparatus (Dionex, San Jose, CA). The chromatographic separation was performed on the ACQUITY UPLC T3 column (1.7 μm, 2.1×100 mm) at a flow rate of 0.25 ml/min. The mobile phase consisted of water containing 0.1% formic acids (Solvent A) and acetonitrile containing 0.1% formic acids (Solvent B). Gradient was used with an isocratic elution of 10% Solvent B for the initial 2.5 min, followed by a linear gradient elution of 10-90 % Solvent B from 2.5 min to 3 min, and the column was returned to its starting conditions after 5 min for column balance.

MS detection was performed in the positive ionization mode with multiple reaction monitoring (MRM). The MRM transition of desloratadine was 311.0→258, and corresponding collision energy was 39 V. The MRM transition of internal standard was 383.0→337, and corresponding collision energy was 22 V. Mass spectrometer was operated with the following parameters: spray voltage, 4.0 kV; heated capillary, 300 °C; HESI probe, 350 °C; sheath gas pressure, 40 psi; auxiliary gas pressure,15 psi.

Sixty mice were treated with DLT (20 mg/kg), and blood and brain samples were collected after the administration at 0.13 h, 0.66 h, 1 h, 2 h, 4 h, 8 h, 12 h, 24 h and 36 h. Methanol was used to precipitate protein and extract analyte. DLT concentration in plasma or brain was assessed by LC-MS/MS.

**Results**

***Specific 5HT_2A_R knockdown in the brain by AAV-ePHP-si-5HT_2A_R***- The effective *si-RNA* sequences targeting 5HT_2A_R were screened by RT-PCR and western blot (Figure S2A-D) in microglia and BV2 cells *(F_3,4_=22.47, p=0.0058)*. The knockdown efficacy of AAV-ePHP-*si-5HT_2A_R* against 5HT_2A_R in the brain and other tissues of APP/PS1 mice were assessed after half a month. Western blot results indicated that intravenous injection of AAV-ePHP-*si-5HT_2A_R* notably reduced 5HT_2A_R expression in the brain of APP/PS1 mice but had no effects on other tissues (Figure S2E-F) (*F_7,16_=8.759, p=0.0005*).

Additionally, the level of 5HT_2A_R in the brain was also detected at the age of ten months after behavior test in an attempt to verify the persistence of 5HT_2A_R knockdown. As shown in Figure S2 G and I, intravenous injection of AAV-ePHP-*si-5HT_2A_R* reduced 5HT_2A_R expression in the brain of APP/PS1 mice at the age of ten months *(F_3,6_=38.29, p<0.0001)*. Notably, the knockdown efficacy of AAV-ePHP-*si-5HT_2A_R* against 5HT_2A_R in different cell populations of the brain was also detected by immunofluorescence assay. As shown in Figure S2H and J, the levels of 5HT_2A_R in neuron, microglia and astrocyte were reduced to 24.8±9.6%, 31.9±3.6% and 27.7±4.6%, respectively *(F_6,7_=80.6, p<0.0001)* (Robin et al., 2018) (Fang et al., 2019). Moreover, no significant difference was determined in the knockdown efficiency of AAV si-5HT_2A_R among neurons, microglia and astrocytes.

***5HT_2A_R antagonism slightly improved anxiety-related behavior in APP/PS1 mice****-* We also detected whether 5HT_2A_R antagonism altered anxiety- and depression-related behaviors in APP/PS1 mice through Open-field test and Forced swimming test, respectively. The results indicated that antagonism of 5HT_2A_R could slightly improve the anxiety-related behaviors in APP/PS1 mice (Figure S3A-B) *(F_3,21_=0.8902, p=0.4624) (F_3,21_=2.241, p=0.6191)*, but had no effects on depression-related behavior in APP/PS1 mice (Figure S3D). This negative result might be also related to the decline of the mice in exercise capacity caused by aging.

***5HT_2A_R knockdown upregulated microglial TLR2/4 in APP/PS1 mice-*** *A*n immunofluorescence assay was performed to investigate TLR2/4 regulation in the microglia lacking 5HT_2A_R. As shown in Figure S5B and C, TLR2/4 was upregulated in microglia with 5HT_2A_R knockdown *(F_5,24_=92.83, p<0.0001)*. In addition, we isolated the populations of microglia that were or were not impacted by *si*-5HT_2A_R and compared the expression of TLR2/4 between these separated populations. In the assay, the microglia in the APP/PS1 mice treated with or without AAV-si-5HT_2A_R were labeled by Iba1 antibody, and 5HT_2A_R level of single microglia in AAV-si-5HT_2A_R-treated APP/PS1 mice in each image was detected and compared with that in APP/PS1 mice, then the single microglia with normal or low expression of 5HT_2A_R in each image were distinguished and their TLR2/4 expression levels were detected (image, n = 12; cell, n = 12).

As shown in Figure S5D and E, compared with the microglia with normal 5HT_2A_R expression (which was not impacted by AAV-si-5HT_2A_R treatment), the microglia with low 5HT_2A_R expression induced by AAV-si-5HT_2A_R treatment exhibited higher level of TLR2/4 (*F_1,66_=131.4, p<0.0001*). These results thereby demonstrated that antagonizing 5HT_2A_R by AAV-si-5HT_2A_R treatment upregulated TLR2/4 expression in microglia of APP/PS1 mice.

***DLT ameliorated AD-like pathology independent of targeting H1 receptor***- Considering that DLT was ever reported as an H1 receptor (H1R) antagonist (Chen et al., 2015), we also examined whether DLT ameliorated AD-like pathology by targeting H1 receptor. For this purpose, related assay with si-RNA of H1 receptor was performed. As shown in Figure S7 A-G, H1R knockdown failed to affect the regulation of DLT against phagocytosis *(F_24,72_=2.972, p=0.0002)*, autophagy or inflammatory events *(F_5,12_=12.82, p=0.0047) (F_5,12_=120.6, p<0.0001)*, which thus implied that DLT ameliorated AD-like pathology independent of H1R targeting.

***DLT suppressed inflammation independent of GR activation*-** Given that GR activation may mediate anti-inflammatory or pro-inflammatory effects in different pathological conditions (Duque Ede & Munhoz, 2016; Oh et al., 2017), we investigated whether GR activation also participated in 5HT_2A_R antagonism-mediated anti-inflammatory effect. As shown in Figure S9A-B, co-treatment of Mifeprex had no effects on 5HT_2A_R antagonism-induced inhibition against inflammatory cytokines TNF-α and IL-6, which thus indicated that GR activation was not involved in 5HT_2A_R antagonism-mediated anti-inflammatory regulation *(F_9,20_=11.87, p=0.0384) (F_9,20_=4.505, p=0.0358)*.

***DLT functioned as a selective 5HT_2A_R antagonist****-* Given the highly structural similarity within 5HT receptor subtypes (Zhang & Stackman, 2015), we detected whether DLT functioned in microglia by specifically targeting 5HT_2A_R receptor. In the assay, p-CREB as an indicator was investigated by western blot based on its potent node in DLT-mediated pathway. In the assay, the known antagonists of 5HT_1A_R (Flopropione) (Burns et al., 2015), 5HT_1D_R (LY310762) (Yang, Huang, Xu, & Duan, 2017), 5HT_2A_R (Ketanserin) (Liu et al., 2013), 5HT_2B_R (PRX08006) (Svejda et al., 2010), 5HT_2C_R (Puerarin) (Pan, Wang, Pu, Yao, & Wang, 2015), 5HT_3_R (Tropisetron) (Yang et al., 2017), 5HT_4_R (Piboserod) (Kjekshus et al., 2009), 5HT_6_R (SB-742457) (Parker et al., 2015) and 5HT_7_R (SB-269970) (Lim, Chess-Williams, & Sellers, 2018) were applied in BV2 cells. The cells were pre-treated with different 5HTR antagonists for 6h, and then exposed to o-Aβ, followed by treatment of DLT. Western blot results demonstrated that 5HT_2A_R antagonist Ketanserin synchronously reversed p-CREB level and pre-treatment of Ketanserin abolished the activity of DLT in stimulating CREB phosphorylation. Notably, pre-treatment of other 5HTR antagonists (except Ketanserin) had no effects on DLT-induced CREB phosphorylation (Figure S10A-F) *(F_8,18_=98.55， p<0.0001) (F_8,18_=257.5, p<0.0001) (F_8,18_=144.0, p<0.0001)*.

In addition, in an attempt to exclude the possibility of monoamine receptors as also the targets of DLT (Nagatomo, Rashid, Abul Muntasir, & Komiyama, 2004), monoamine receptor inhibitors were applied. As shown in Figure S10G-H, Western blot results demonstrated that none of α-adrenoceptor inhibitor Phentolamine, β-adrenoceptor inhibitor Propranolol (Dobarro, Gerenu, & Ramirez, 2013) or Dopamine receptor inhibitor Chlorprothixene (Skov, Johansen, & Linnet, 2015) had effects on DLT-induced CREB phosphorylation (Figure S10G-H) *(F_8,18_=37.78, p<0.0001)*.

Thus, all results implied that DLT functions in microglia through specifically targeting 5HT_2A_R receptor.

***DLT dose-dependently restrained the progression of AD phenotypes by antagonizing 5HT_2A_R-*** As shown in Figure S11A-F, several endpoints (Aβ of phagocytosis *(F_3,32_=0.8413, p=0.4814)* and degradation *(F_3,156_=13.74, p<0.0001)* as well as anti-inflammatory effects *(F_4,5_=12.32, p=0.0001) (F_4,5_=124.8, p=0.0269)* were examined using different doses of DLT (2, 5 and 10 μM), and the results demonstrated that DLT dose-dependently restrained the progression of AD-like phenotypes by antagonizing 5HT_2A_R.

***DLT could pass through the blood-brain barrier in mice-*** DLT concentrations in plasma and brain were assessed in C57BL/6 mice treated with 20 mg/kg of DLT. As shown in Figure S12A-B, the mean time to maximum plasma concentrations (Tmax) occurred at approximately 2 hours post dose and mean steady state peak plasma concentrations (Cmax) and AUC of 4.147 μg/mL and 60.68 μg/mL were observed, respectively. These results thus indicated the good oral availability of DLT. Additionally, mean time to Tmax in brain occurred at approximately 8 hours post dose and brain Cmax and AUC of 1.328 μg/g and 28.94 μg/g were observed, respectively. Notably, the blood brain ratio of DLT (AUC_brain_ / AUC_blood_) is 0.476, indicating a good BBB penetrance of DLT.

***DLT reduced amyloid-plaque and promoted phagocytosis and autophagy in the cortex of APP/PS1 mice-*** Given the potential non-specificities against different brain tissue populations for AAV.ePHP-*si-5HT_2A_R* and DLT, several endpoints such as senile plaque, Aβ phagocytosis and autophagy marker protein LC3 were also detected in the cortex of the brain (Figure S13A-F). All results indicated that DLT reduced amyloid-plaque *(F_3,9_=19.51, p=0.0003)* and promoted phagocytosis *(F_3,6_=40.98, p=0.0002)* and autophagy *(F_3,9_=7.079, p=0.0096)*, verifying the global beneficial effects of DLT.

***DLT protected against Aβ-induced apoptosis in primary neuron-*** We performed the related assays to investigate the probable effects of DLT on neurons (non-microglia populations). As shown in Fig. S14A-E, treatment of DLT attenuated Aβ-induced cell viability repression *(F_4,12_=4.045, p=0.0265)*, cell apoptosis and caspase3-dependent apoptotic pathway activation *(F_4,30_=10.40, p<0.0001) (F_4,40_=135.6, p<0.0001)* in primary neurons. These results thereby demonstrated that DLT function also beneficial effects on non-microglia populations.

**References and notes:**

Burns, S. M., Vetere, A., Walpita, D., Dancik, V., Khodier, C., Perez, J., . . . Altshuler, D. (2015). High-throughput luminescent reporter of insulin secretion for discovering regulators of pancreatic Beta-cell function. *Cell Metab, 21*(1), 126-137. doi:10.1016/j.cmet.2014.12.010

Chen, M., Xu, S., Zhou, P., He, G., Jie, Q., & Wu, Y. (2015). Desloratadine citrate disodium injection, a potent histamine H(1) receptor antagonist, inhibits chemokine production in ovalbumin-induced allergic rhinitis guinea pig model and histamine-induced human nasal epithelial cells via inhibiting the ERK1/2 and NF-kappa B signal cascades. *Eur J Pharmacol, 767*, 98-107. doi:10.1016/j.ejphar.2015.10.014

Dobarro, M., Gerenu, G., & Ramirez, M. J. (2013). Propranolol reduces cognitive deficits, amyloid and tau pathology in Alzheimer's transgenic mice. *Int J Neuropsychopharmacol, 16*(10), 2245-2257. doi:10.1017/S1461145713000631

Duque Ede, A., & Munhoz, C. D. (2016). The Pro-inflammatory Effects of Glucocorticoids in the Brain. *Front Endocrinol (Lausanne), 7*, 78. doi:10.3389/fendo.2016.00078

Fang, E. F., Hou, Y., Palikaras, K., Adriaanse, B. A., Kerr, J. S., Yang, B., . . . Bohr, V. A. (2019). Mitophagy inhibits amyloid-beta and tau pathology and reverses cognitive deficits in models of Alzheimer's disease. *Nat Neurosci, 22*(3), 401-412. doi:10.1038/s41593-018-0332-9

Guo, X., Lv, J., Lu, J., Fan, L., Huang, X., Hu, L., . . . Shen, X. (2018). Protopanaxadiol derivative DDPU improves behavior and cognitive deficit in AD mice involving regulation of both ER stress and autophagy. *Neuropharmacology, 130*, 77-91. doi:10.1016/j.neuropharm.2017.11.033

Guo, X. D., Sun, G. L., Zhou, T. T., Xu, X., Zhu, Z. Y., Rukachaisirikul, V., . . . Shen, X. (2016). Small molecule LX2343 ameliorates cognitive deficits in AD model mice by targeting both amyloid beta production and clearance. *Acta Pharmacol Sin, 37*(10), 1281-1297. doi:10.1038/aps.2016.80

Ising, C., Venegas, C., Zhang, S., Scheiblich, H., Schmidt, S. V., Vieira-Saecker, A., . . . Heneka, M. T. (2019). NLRP3 inflammasome activation drives tau pathology. *Nature, 575*(7784), 669-673. doi:10.1038/s41586-019-1769-z

Katta, A., Dhananjeyan, M., Bykowski, C., Erhardt, P., Hacker, M., White, D. B., & Bachmann, K. (2007). Verapamil, but not probenecid, co-administration can convert desloratadine to a sedating antihistamine in mice. *Drug Metab Lett, 1*(1), 7-11. doi:10.2174/187231207779814391

Kjekshus, J. K., Torp-Pedersen, C., Gullestad, L., Kober, L., Edvardsen, T., Olsen, I. C., . . . Levy, F. O. (2009). Effect of piboserod, a 5-HT4 serotonin receptor antagonist, on left ventricular function in patients with symptomatic heart failure. *Eur J Heart Fail, 11*(8), 771-778. doi:10.1093/eurjhf/hfp087

Lim, I., Chess-Williams, R., & Sellers, D. (2018). 5-HT2A receptor is the predominant receptor mediating contraction of the isolated porcine distal ureter to 5-HT in young and old animals. *Eur J Pharmacol, 818*, 328-334. doi:10.1016/j.ejphar.2017.11.001

Liu, C., Zhang, X., Zhou, J. X., Wei, W., Liu, D. H., Ke, P., . . . Su, D. F. (2013). The protective action of ketanserin against lipopolysaccharide-induced shock in mice is mediated by inhibiting inducible NO synthase expression via the MEK/ERK pathway. *Free Radic Biol Med, 65*, 658-666. doi:10.1016/j.freeradbiomed.2013.07.045

Minkeviciene, R., Rheims, S., Dobszay, M. B., Zilberter, M., Hartikainen, J., Fulop, L., . . . Tanila, H. (2009). Amyloid beta-induced neuronal hyperexcitability triggers progressive epilepsy. *J Neurosci, 29*(11), 3453-3462. doi:10.1523/JNEUROSCI.5215-08.2009

Nagatomo, T., Rashid, M., Abul Muntasir, H., & Komiyama, T. (2004). Functions of 5-HT2A receptor and its antagonists in the cardiovascular system. *Pharmacol Ther, 104*(1), 59-81. doi:10.1016/j.pharmthera.2004.08.005

Oh, K. S., Patel, H., Gottschalk, R. A., Lee, W. S., Baek, S., Fraser, I. D. C., . . . Sung, M. H. (2017). Anti-Inflammatory Chromatinscape Suggests Alternative Mechanisms of Glucocorticoid Receptor Action. *Immunity, 47*(2), 298-309 e295. doi:10.1016/j.immuni.2017.07.012

Pan, X., Wang, J., Pu, Y., Yao, J., & Wang, H. (2015). Effect of Puerarin on Expression of ICAM-1 and TNF-alpha in Kidneys of Diabetic Rats. *Med Sci Monit, 21*, 2134-2140. doi:10.12659/MSM.893714

Parker, C. A., Rabiner, E. A., Gunn, R. N., Searle, G., Martarello, L., Comley, R. A., . . . Cunningham, V. J. (2015). Human Kinetic Modeling of the 5HT6 PET Radioligand 11C-GSK215083 and Its Utility for Determining Occupancy at Both 5HT6 and 5HT2A Receptors by SB742457 as a Potential Therapeutic Mechanism of Action in Alzheimer Disease. *J Nucl Med, 56*(12), 1901-1909. doi:10.2967/jnumed.115.162743

Robin, L. M., Oliveira da Cruz, J. F., Langlais, V. C., Martin-Fernandez, M., Metna-Laurent, M., Busquets-Garcia, A., . . . Marsicano, G. (2018). Astroglial CB1 Receptors Determine Synaptic D-Serine Availability to Enable Recognition Memory. *Neuron, 98*(5), 935-944 e935. doi:10.1016/j.neuron.2018.04.034

Skov, L., Johansen, S. S., & Linnet, K. (2015). Postmortem femoral blood reference concentrations of aripiprazole, chlorprothixene, and quetiapine. *J Anal Toxicol, 39*(1), 41-44. doi:10.1093/jat/bku121

Souchet, B., Audrain, M., Billard, J. M., Dairou, J., Fol, R., Orefice, N. S., . . . Cartier, N. (2019). Inhibition of DYRK1A proteolysis modifies its kinase specificity and rescues Alzheimer phenotype in APP/PS1 mice. *Acta Neuropathol Commun, 7*(1), 46. doi:10.1186/s40478-019-0678-6

Svejda, B., Kidd, M., Giovinazzo, F., Eltawil, K., Gustafsson, B. I., Pfragner, R., & Modlin, I. M. (2010). The 5-HT(2B) receptor plays a key regulatory role in both neuroendocrine tumor cell proliferation and the modulation of the fibroblast component of the neoplastic microenvironment. *Cancer, 116*(12), 2902-2912. doi:10.1002/cncr.25049

Teixeira, C. M., Rosen, Z. B., Suri, D., Sun, Q., Hersh, M., Sargin, D., . . . Ansorge, M. S. (2018). Hippocampal 5-HT Input Regulates Memory Formation and Schaffer Collateral Excitation. *Neuron, 98*(5), 992-1004 e1004. doi:10.1016/j.neuron.2018.04.030

Xu, X., Shi, X., Chen, Y., Zhou, T., Wang, J., Xu, X., . . . Shen, X. (2018). HS218 as an FXR antagonist suppresses gluconeogenesis by inhibiting FXR binding to PGC-1alpha promoter. *Metabolism, 85*, 126-138. doi:10.1016/j.metabol.2018.03.016

Yang, Y., Huang, H., Xu, Z., & Duan, J. K. (2017). Serotonin and Its Receptor as a New Antioxidant Therapeutic Target for Diabetic Kidney Disease. *J Diabetes Res, 2017*, 7680576. doi:10.1155/2017/7680576

Zhang, G., & Stackman, R. W., Jr. (2015). The role of serotonin 5-HT2A receptors in memory and cognition. *Front Pharmacol, 6*, 225. doi:10.3389/fphar.2015.00225

Zhong, L., Xu, Y., Zhuo, R., Wang, T., Wang, K., Huang, R., . . . Chen, X. F. (2019). Soluble TREM2 ameliorates pathological phenotypes by modulating microglial functions in an Alzheimer's disease model. *Nat Commun, 10*(1), 1365. doi:10.1038/s41467-019-09118-9

Zhong, Q., Yu, H., Huang, C., Zhong, J., Wang, H., Xu, J., & Cheng, Y. (2019). FCPR16, a novel phosphodiesterase 4 inhibitor, produces an antidepressant-like effect in mice exposed to chronic unpredictable mild stress. *Prog Neuropsychopharmacol Biol Psychiatry, 90*, 62-75. doi:10.1016/j.pnpbp.2018.10.017

Zhou, C., Zhong, W., Zhou, J., Sheng, F., Fang, Z., Wei, Y., . . . Lin, J. (2012). Monitoring autophagic flux by an improved tandem fluorescent-tagged LC3 (mTagRFP-mWasabi-LC3) reveals that high-dose rapamycin impairs autophagic flux in cancer cells. *Autophagy, 8*(8), 1215-1226. doi:10.4161/auto.20284

**Figure legends**

**Figure S1. DLT inhibited 5HT_2A_R in primary neurons and microglia. (A)** Calcium

assay results indicated that DLT dose-dependently inhibited 5HT_2A_R in primary neurons in the presence of 10 μM of 5HT (IC_50_ = 9.177 μM). Knockdown of 5HT_2A_R deprived DLT of its capability in regulating calcium flow in primary neurons. **(B)** Calcium assay results indicated that DLT dose-dependently inhibited 5HT_2A_R in primary microglia in the presence of 10 μM of 5HT (IC_50_ = 0.3185 μM). Knockdown of 5HT_2A_R deprived DLT of its capability in regulating calcium flow in primary microglia.

**Figure S2. Specific 5HT_2A_R knockdown in the brain by AAV-ePHP-*si-5HT_2A_R*.** (**A, B**) Effective *si-RNA* sequences targeting 5HT_2A_R were screened by RT-PCR in primary microglia and BV2 cells (n = 3). **(C)** Western blot assay and **(D)** its quantification results demonstrated that the protein level of 5HT_2A_R was reduced by about 50% after *siRNA* transfection (n = 2). **(E, F)** The efficacy of 5HT_2A_R knockdown in the brain and other tissues of mice were assessed after half a month. **(E)** western blot assay and **(F)** its quantification results demonstrated that intravenous injection of AAV-ePHP-*si-5HT_2A_R* notably reduced the expression of 5HT_2A_R in the cortex and hippocampus of APP/PS1 mice but had no effects on other tissues (n = 3). **(G, I)** Efficacy of 5HT_2A_R knockdown in the brain of mice was assessed at the age of ten months. (**G**) Immunofluorescence assay and (**I**) its quantification demonstrated that intravenous injection of AAV-ePHP-*si-5HT_2A_R* reduced the expressions of 5HT_2A_R in the cortex and hippocampus of APP/PS1 mice at the age of ten months (n = 3). Scale bar: 200 µm. (**H, J**) Knockdown efficacies of AAV-ePHP-*si-5HT_2A_R* against 5HT_2A_R in different cell populations of brain were also detected by immunofluorescence assay. (**H**) Immunofluorescence assay and (**J**) its quantification demonstrated that the levels of 5HT_2A_R in neurons, microglia and astrocytes were reduced by 24.8±9.6%, 31.9±3.6% and 27.7±4.6%, respectively (n = 3). Scale bar: 50 µm. All values of animal experiments were presented as mean ± SEM. For animal tissue assay, ^*^P < 0.05, ^**^P < 0.01, ^***^P < 0.001 compared with APP/PS1 group by t-test. For cell assay, *P < 0.05 compared with *si-Ctrl* by One-way ANOVA*.*

**Figure S3. 5HT_2A_R antagonism slightly improved anxiety-related behavior in APP/PS1 mice. (A, B)** The results of Open-field test indicated that treatment of DLT or AAV-*si-5HT_2A_R* slightly improved the anxiety-related behaviors in APP/PS1 mice (n = 8). **(C)** Swimming speed assay results indicated that there was no difference between each group (n = 8). **(D)** Forced swimming assay results indicated that treatment of DLT or AAV-*si-5HT_2A_R* had no effects on depression-related behaviors in APP/PS1 mice (n = 8). **(E)** Quantification results of Figure 1M demonstrated that treatment of DLT or AAV-*si-5HT_2A_R* reversed the suppression of synapse-related proteins, including PSD95, synaptophysin and VAMP2 in the brains of APP/PS1 mice (n = 4). All values of animal experiments were presented as mean ± SEM. For animal tissue assays, ^#^P < 0.05compared with WT group by t-test. *P < 0.05 compared with APP/PS1 group by Two-way ANOVA.

**Figure S4. DLT suppressed Aβ level independent of amyloidogenic pathways***.* **(A)** Quantification results of Figure 2E demonstrated that no significant difference was determined in APP protein level between DLT-treated AAV-*si-5HT_2A_R* injected APP/PS1 mice and vehicle-treated AAV-*si-5HT_2A_R* injected APP/PS1 mice (n = 4). (**B**) Jess assay and (**C**) its quantification demonstrated that treatment of DLT or AAV-*si*-*5HT_2A_R* rendered no influences on BACE1, sAPPβ and p-APP (n = 4). **(D)** Quantification results of Figure S4B demonstrated that treatment of DLT or AAV-*si*-*5HT_2A_R* rendered no influences on BACE1, sAPPβ or p-APP (n = 4). All values of animal experiments were presented as mean ± SEM. For animal tissue assays, ^###^P < 0.001 compared with WT group by t-test.

**Figure S5. 5HT_2A_R knockdown upregulated microglial TLR2/4 in APP/PS1 mice. (A)** Quantification results of Figure 2M demonstrated that treatment of DLT or AAV-*si-5HT_2A_R* increased TLR2/4 protein levels in the brain of APP/PS1 mice (n = 4). **(B)** Immunofluorescence assay and **(C)** its quantification demonstrated that TLR2/4 were upregulated in 5HT_2A_ knockdown microglia (n = 4). Scale bar: 50 µm. **(D)** Immunofluorescence assay and **(E)** its quantification demonstrated that antagonizing 5HT_2A_R by AAV-si-5HT_2A_R treatment upregulated TLR2/4 in microglia (image, n = 12; cell, n = 12). Scale bar: 20 µm. All values of animal experiments were presented as mean ± SEM. For animal tissue assays, ^#^P < 0.05 compared with APP/PS1 group by Two-way ANOVA.

**Figure S6. DLT treatment repressed microglial neuroinflammation in APP/PS1 mice by antagonizing 5HT_2A_R. (A-D)** The results of ELISA and RT-PCR demonstrated that treatment of DLT or AAV-*si-5HT_2A_R* reduced the protein and mRNA levels of IL-4 and IL-10 in the brain of APP/PS1 mice (n = 6). All values of animal experiments were presented as mean ± SEM. For animal tissue assays, ^#^P < 0.05, ^##^P < 0.01 compared with WT group by t-test. *P < 0.05, **P < 0.01, ***P < 0.001 compared with APP/PS1 group by Two-way ANOVA.

**Figure S7. DLT ameliorated AD-like pathology independent of H1 receptor targeting. (A, B)** Efficacy of H1R knockdown in microglia was assessed by western blot. **(C)** Real-time detection of live cell imaging assay and **(D)** its quantifications (levels of Aβ phagocytosis within four hours) demonstrated that H1R knockdown had no effects on the DLT-induced phagocytosis promotion. **(E)** Autophagy flux assay results demonstrated that H1R knockdown had no effects on the DLT-induced autophagy stimulation. Scale bar: 10 µm. (**F, G**) RT-PCR results demonstrated that H1R knockdown had no effects on the DLT-induced anti-inflammatory effects. All values of experiments were presented as mean ± SEM. For cell assays, ^###^P < 0.05 compared with *si-Ctrl or si-H1R* by One-way ANOVA*. **P < 0.05, ****P < 0.01, *****P < 0.005 compared with *si-Ctrl+o-Aβ or si-H1R+o-Aβ* by One-way ANOVA*.*

**Figure S8. DLT treatment promoted autophagy and repressed inflammation in APP/PS1 mice through 5-HT_2A_R/cAMP/PKA/CREB/Sirt1 signaling pathway. (A)** Quantification results of Figure 5C demonstrated that treatment of DLT or AAV-*si-5HT_2A_R* increased the protein level of Sirt1 in the brains of APP/PS1 mice (n = 4)*.* **(B)** RT-PCR results demonstrated that treatment of DLT or AAV-*si-5HT_2A_R* upregulated mRNA level of Sirt1 in APP/PS1 mice*.* All values of experiments were presented as mean ± SEM. For animal tissue assays, ^##^P < 0.01 compared with WT group by t-test. *P < 0.05 compared with APP/PS1 group by Two-way ANOVA.

**Figure S9. DLT suppressed inflammation independent of GR signaling. (A, B)** RT-PCR results indicated that co-treatment of Mifepr*ex* had no effects on the 5HT_2A_R antagonism-induced inhibition of inflammatory cytokines TNF-α and IL-6 (n = 3). Data were obtained from three independent experiments. ^#^P < 0.05, ^##^P < 0.01 compared with DMSO. *P < 0.05, **P < 0.01 compared with o-Aβ.

**Figure S10. *DLT functioned as a selective 5HT_2A_R antagonist*. (A-H)** BV2 cells were pre-treated with different antagonists of 5-HTR for 6h, and then exposed to o-Aβ, followed by treatment of DLT. The level of phosphorylated CREB was detected by western blot assay. **(A, C, E, G)** Western blot assay and **(B, D, F, H)** its quantification results demonstrated that pre-treatment of Ketanserin abolished the DLT-induced promotion of CREB phosphorylation, while pre-treatment of other 5HTR antagonists (except Ketanserin) or monoamine receptor inhibitors had no effects on the DLT-stimulated CREB phosphorylation (n = 3). ^#^P < 0.05, ^##^P < 0.01 compared with DMSO by One-way ANOVA. *P < 0.05, **P < 0.01 compared with o-Aβ by One-way ANOVA.

**Figure S11. DLT dose-dependently suppressed the progression of AD phenotypes by antagonizing 5HT_2A_R.** **(A-E)** Several endpoints (Aβ phagocytosis and degradation, and anti-inflammatory effects) were examined against different doses of DLT (2, 5 and 10μM). The results demonstrated that DLT dose-dependently suppressed the progression of AD phenotypes by antagonizing 5HT_2A_R. ^#^P < 0.05, ^##^P < 0.01 compared with DMSO by One-way ANOVA. *P < 0.05, **P < 0.01 compared with o-Aβ by One-way ANOVA.

**Figure S12. DLT could pass through the blood-brain barrier. (A, B)** DLT concentrations in plasma and brain were assessed in C57BL/6 mice treated with 20 mg/kg DLT. Shown in the figure were the mean concentrations of DLT in plasma and brain homogenates at each sampling time.

**Figure S13. DLT reduced amyloid-plaque and promoted the processes of phagocytosis and autophagy in the cortex of APP/PS1 mice.** **(A, B)** Thioflavin S staining and its quantification results demonstrated that DLT reduced amyloid-plaque in the cortex of APP/PS1 mice (n = 4). Scale bar: 200 µm. **(C, D)** Immunofluorescence assay and its quantification results demonstrated that DLT promoted Aβ phagocytosis in the cortex of APP/PS1 mice (n = 3). Scale bar: 10 µm. **(E, F)** Immunofluorescence assay and its quantification results demonstrated that DLT promoted autophagy in the cortex of APP/PS1 mice (n = 4). Scale bar: 200 µm. All values of animal experiments were presented as mean ± SEM. For animal tissue assays, ^###^P < 0.05 compared with WT group by t-test. **P < 0.01, ***P < 0.001 compared with APP/PS1 group by Two-way ANOVA.

**Figure S14. DLT protected against Aβ-induced apoptosis in primary neurons. (A)** DLT attenuated the cell viability reduction induced by Aβ in primary neurons (n = 4). **(B)** DLT attenuated neuronal apoptosis induced by Aβ in primary neurons (n = 4). **(C)** Western blot and its quantification results **(D, E)** demonstrated that DLT attenuated the activation of caspase3-dependent apoptotic pathway (n = 3). All values of experiments were presented as mean ± SEM. ^#^P < 0.05, ^##^P < 0.01, ^###^P < 0.001 compared with DMSO One-way ANOVA. *P < 0.05, **P < 0.01, ***P < 0.001 compared with o-Aβ_25-35_ by One-way ANOVA.
