# Supplementary figures and images for: Antiallergic drug desloratadine as a selective antagonist of 5HT2A receptor ameliorates pathology of Alzheimer's disease model mice by improving microglial dysfunction
Source: Aging Cell. 2020 Dec 24;20(1):e13286. doi: 10.1111/acel.13286 (PMC7811850; doi:10.1111/acel.13286)

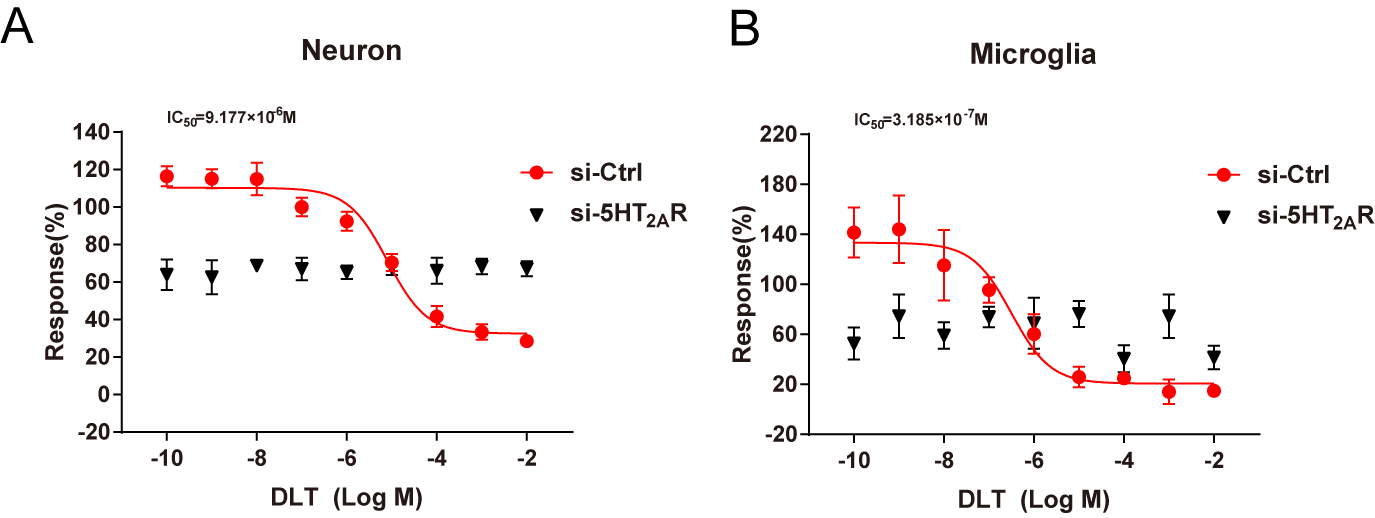

Supplement: Supplementary file 2 — Fig S1 [file ACEL-20-e13286-s002.tif]

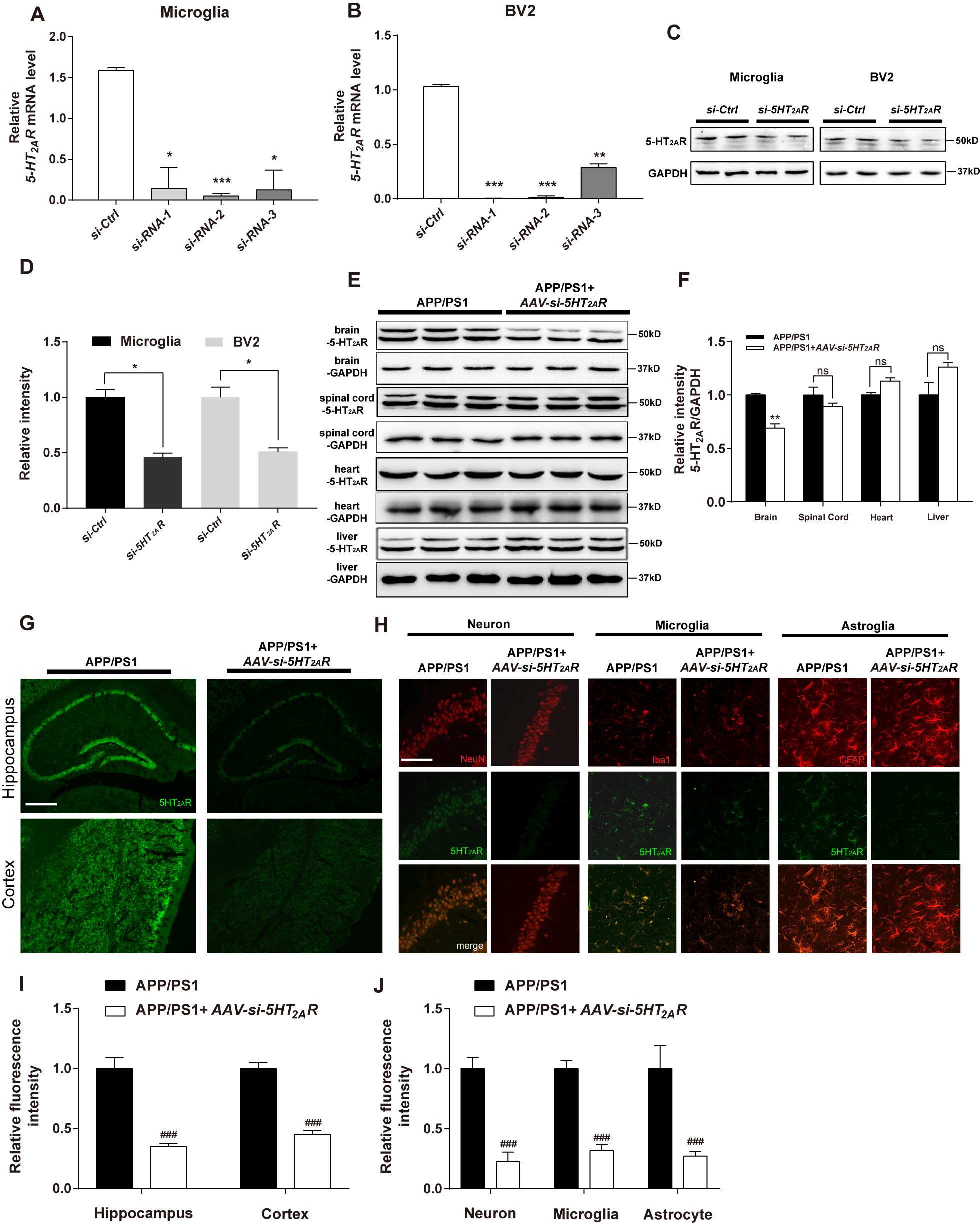

Supplement: Supplementary file 3 — Fig S2 [file ACEL-20-e13286-s003.tif]

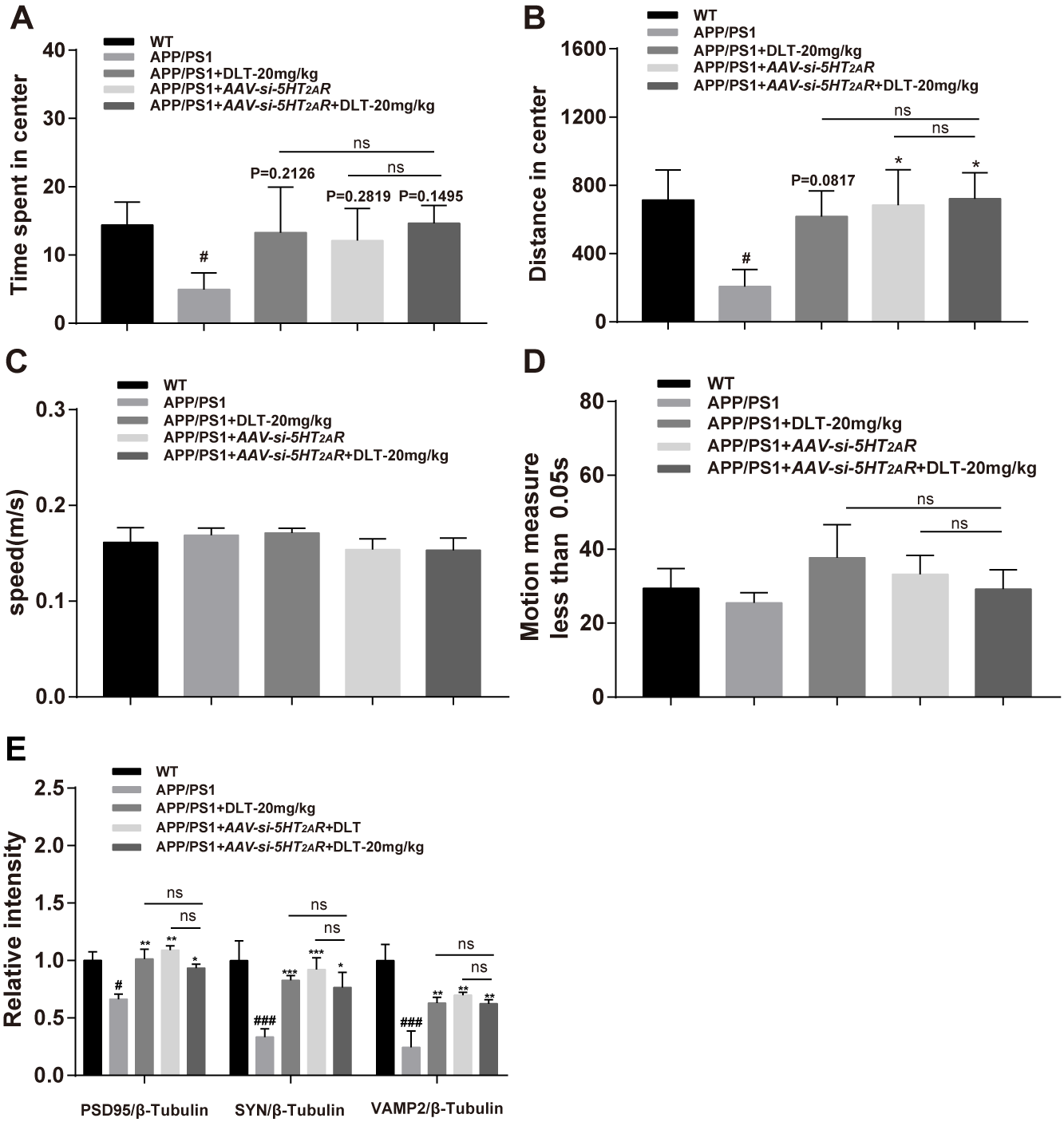

Supplement: Supplementary file 4 — Fig S3 [file ACEL-20-e13286-s004.tif]

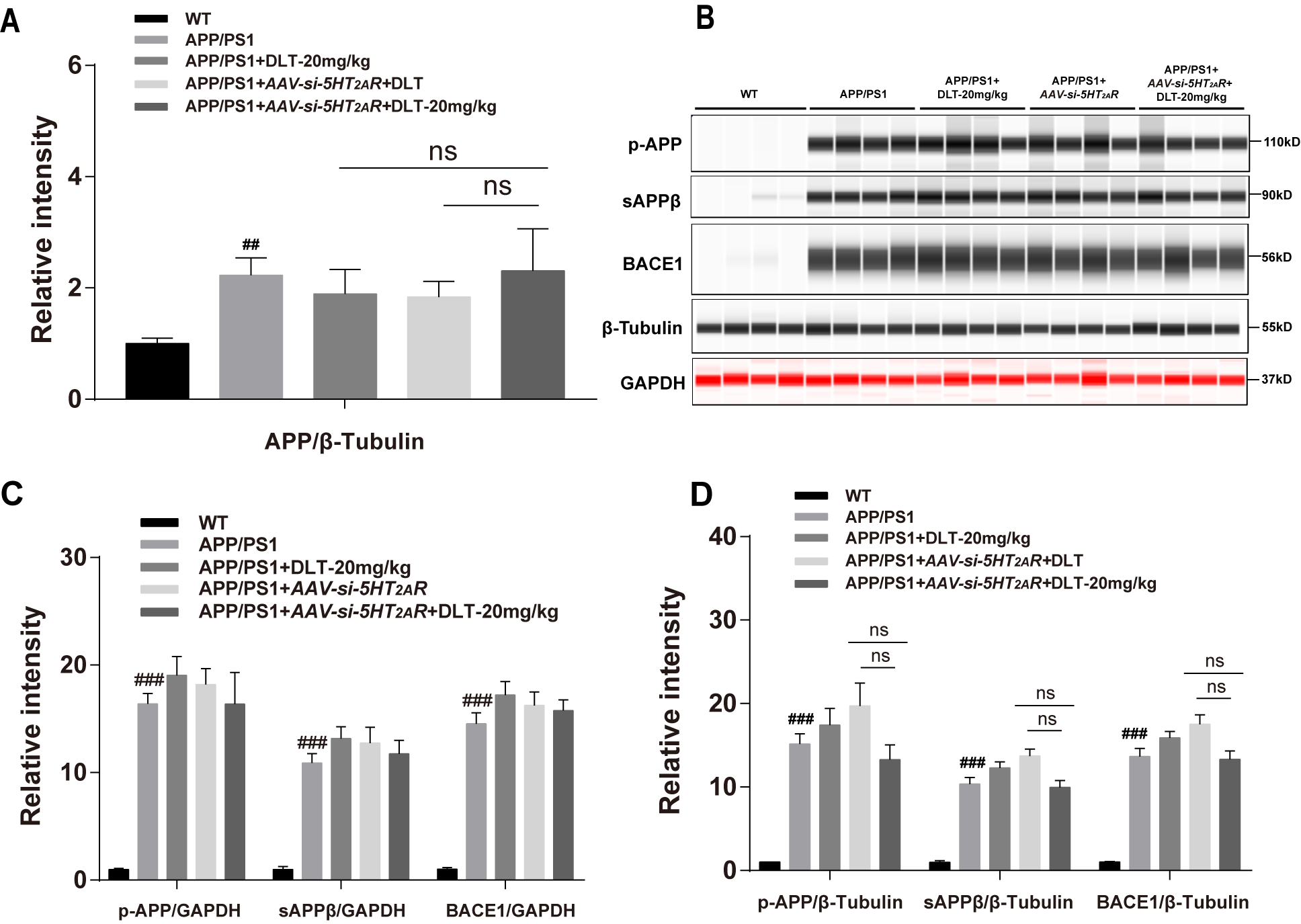

Supplement: Supplementary file 5 — Fig S4 [file ACEL-20-e13286-s005.tif]

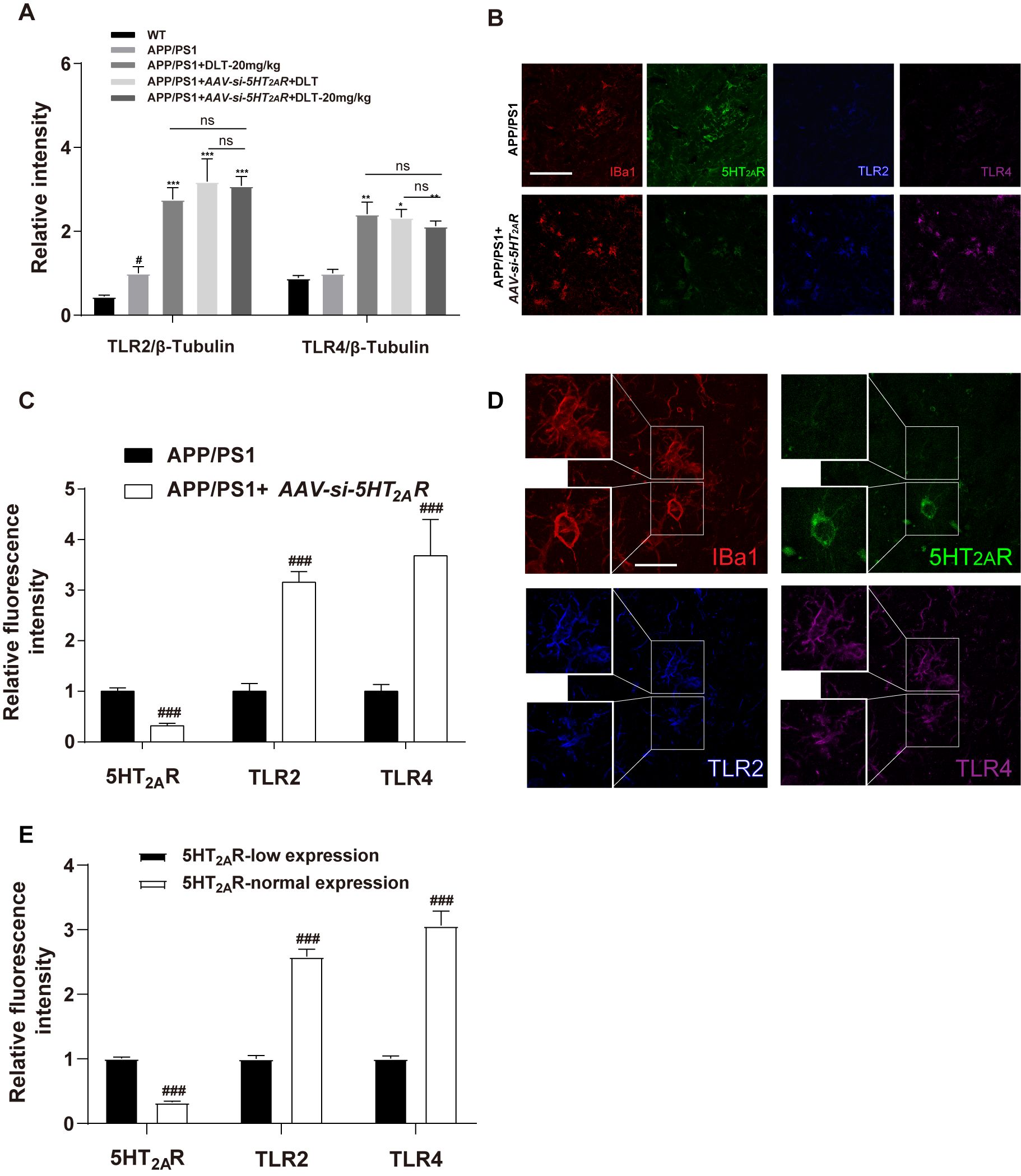

Supplement: Supplementary file 6 — Fig S5 [file ACEL-20-e13286-s006.tif]

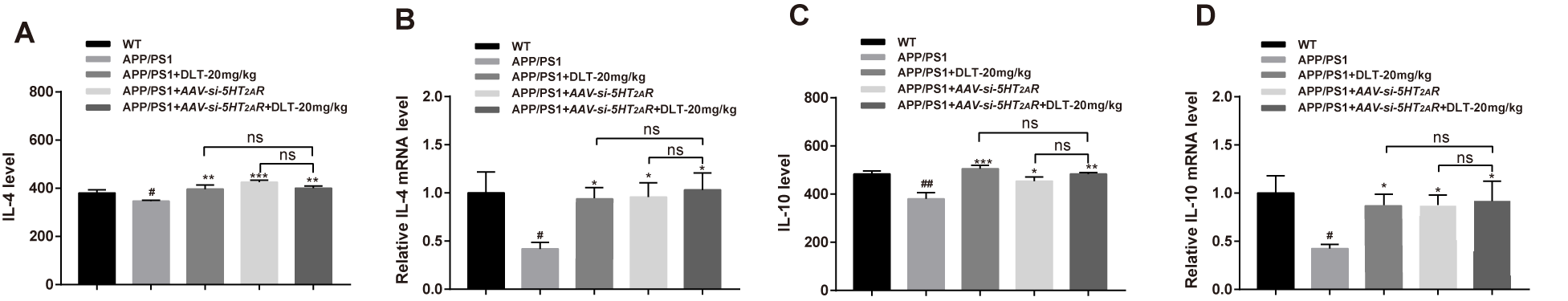

Supplement: Supplementary file 7 — Fig S6 [file ACEL-20-e13286-s007.tif]

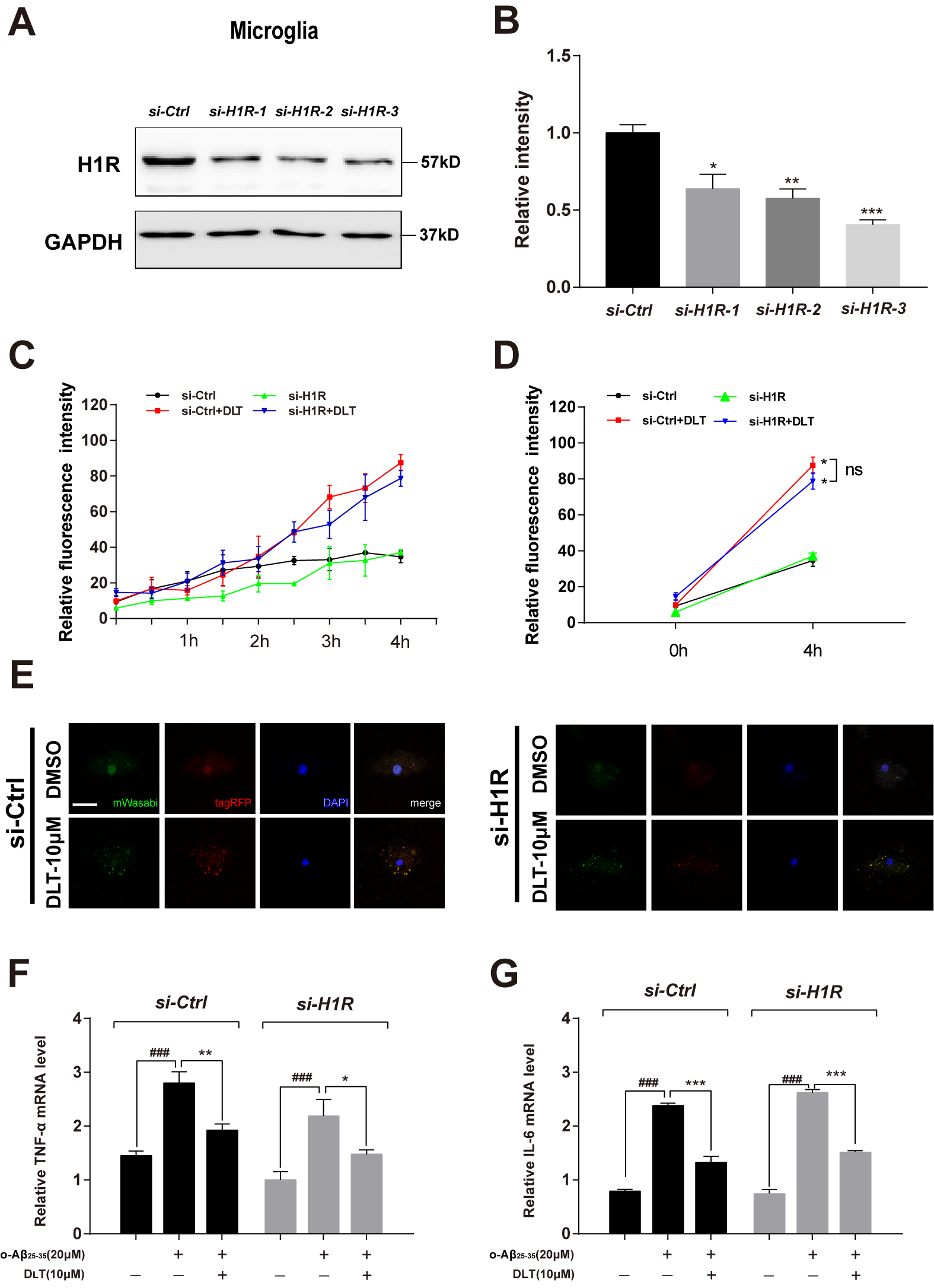

Supplement: Supplementary file 8 — Fig S7 [file ACEL-20-e13286-s008.tif]

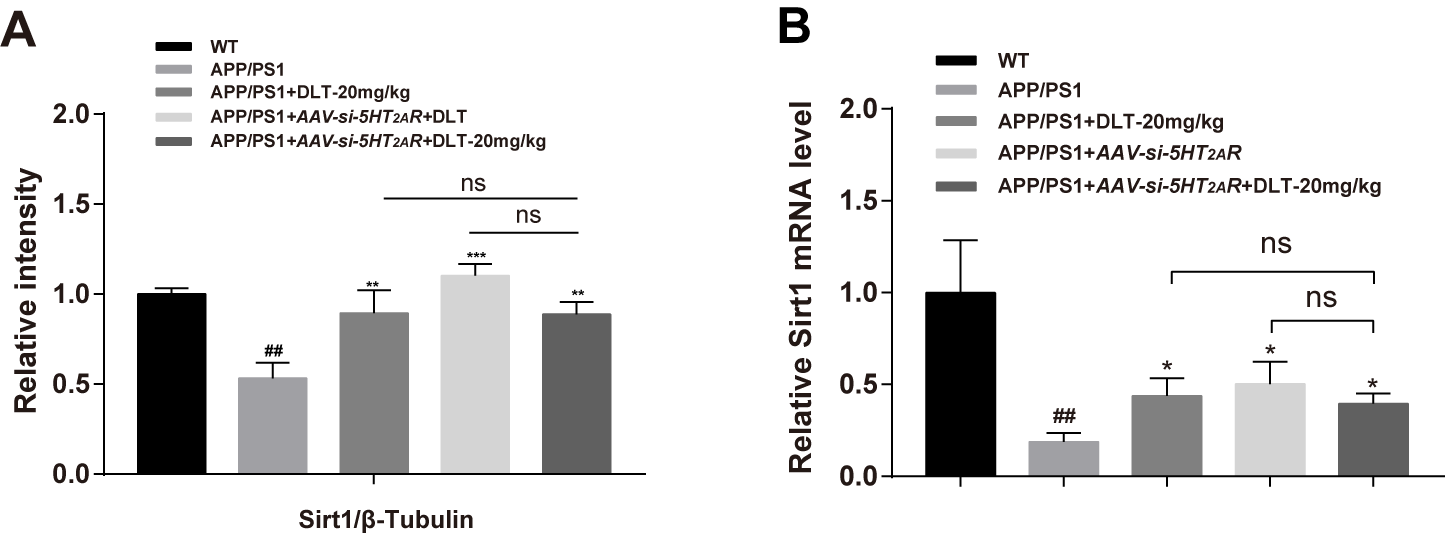

Supplement: Supplementary file 9 — Fig S8 [file ACEL-20-e13286-s009.tif]

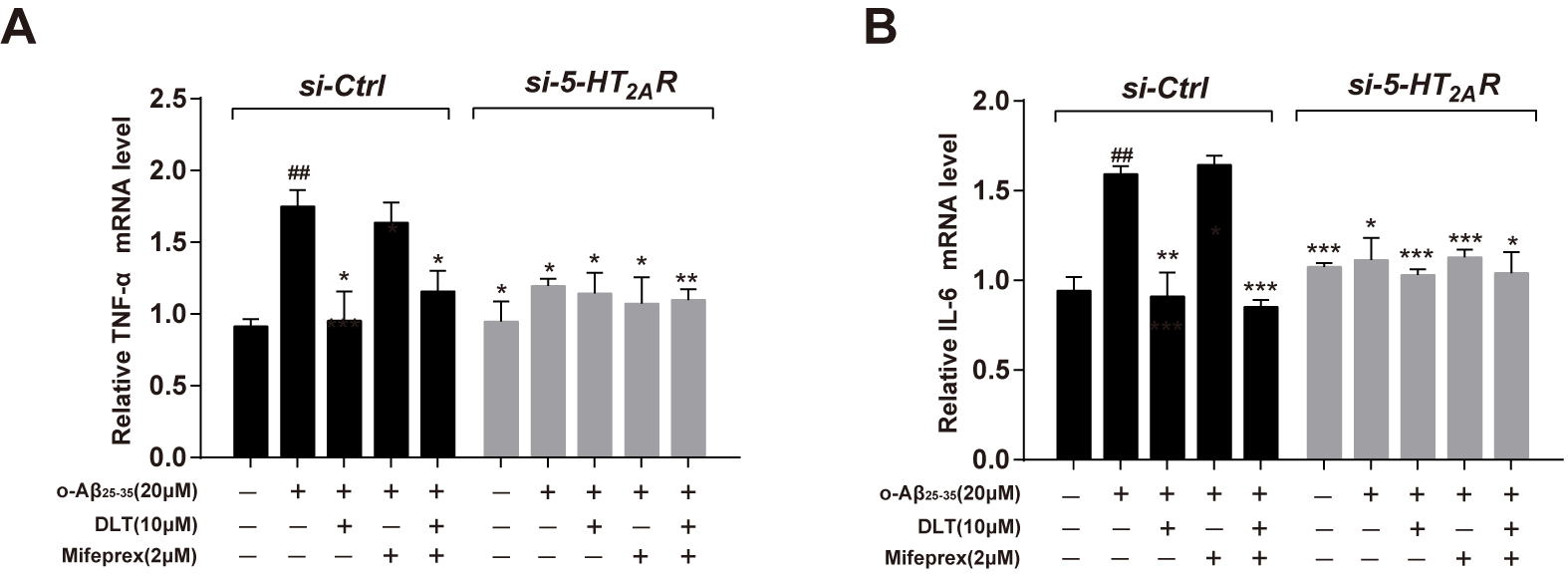

Supplement: Supplementary file 10 — Fig S9 [file ACEL-20-e13286-s010.tif]

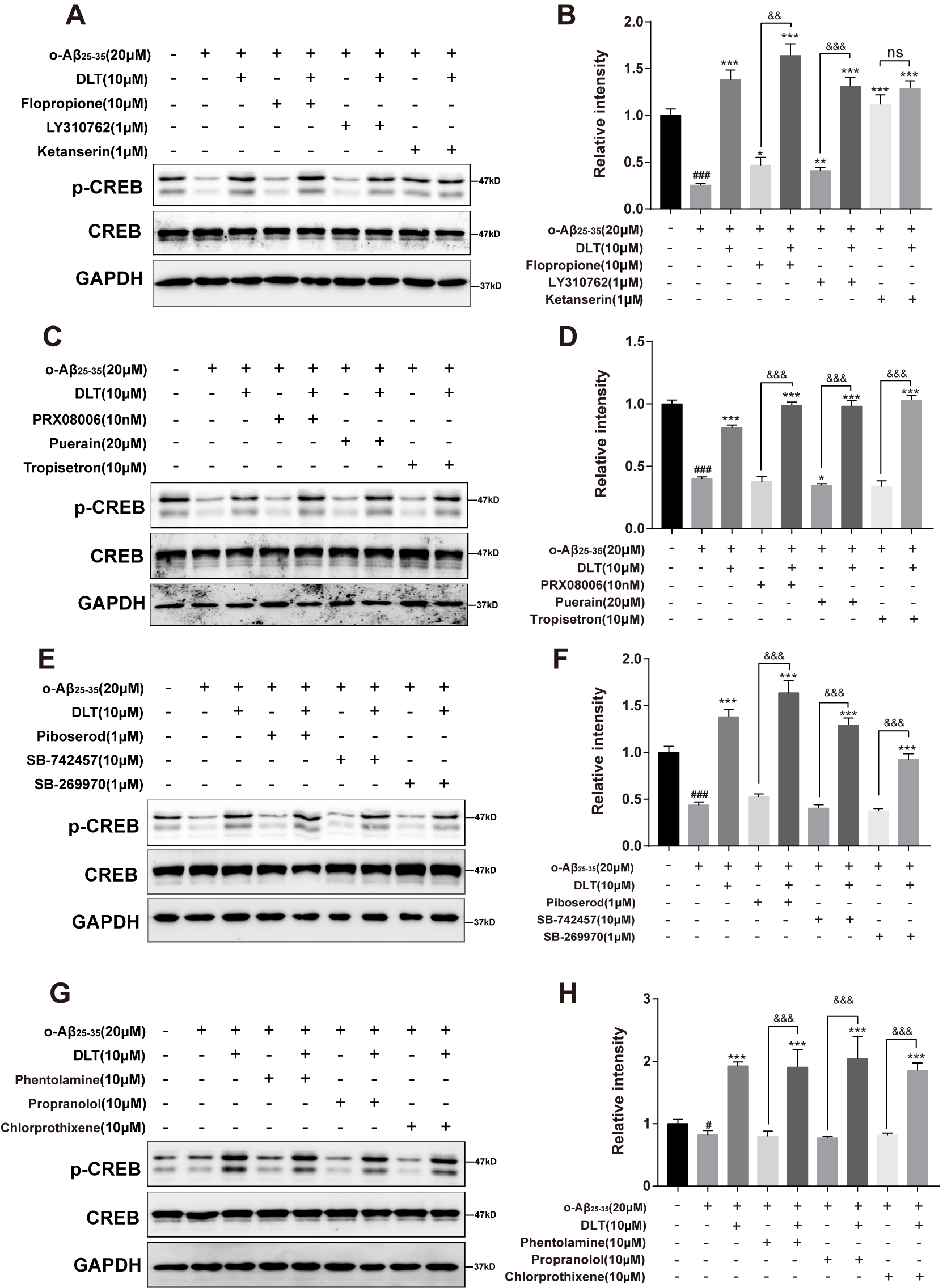

Supplement: Supplementary file 11 — Fig S10 [file ACEL-20-e13286-s011.tif]

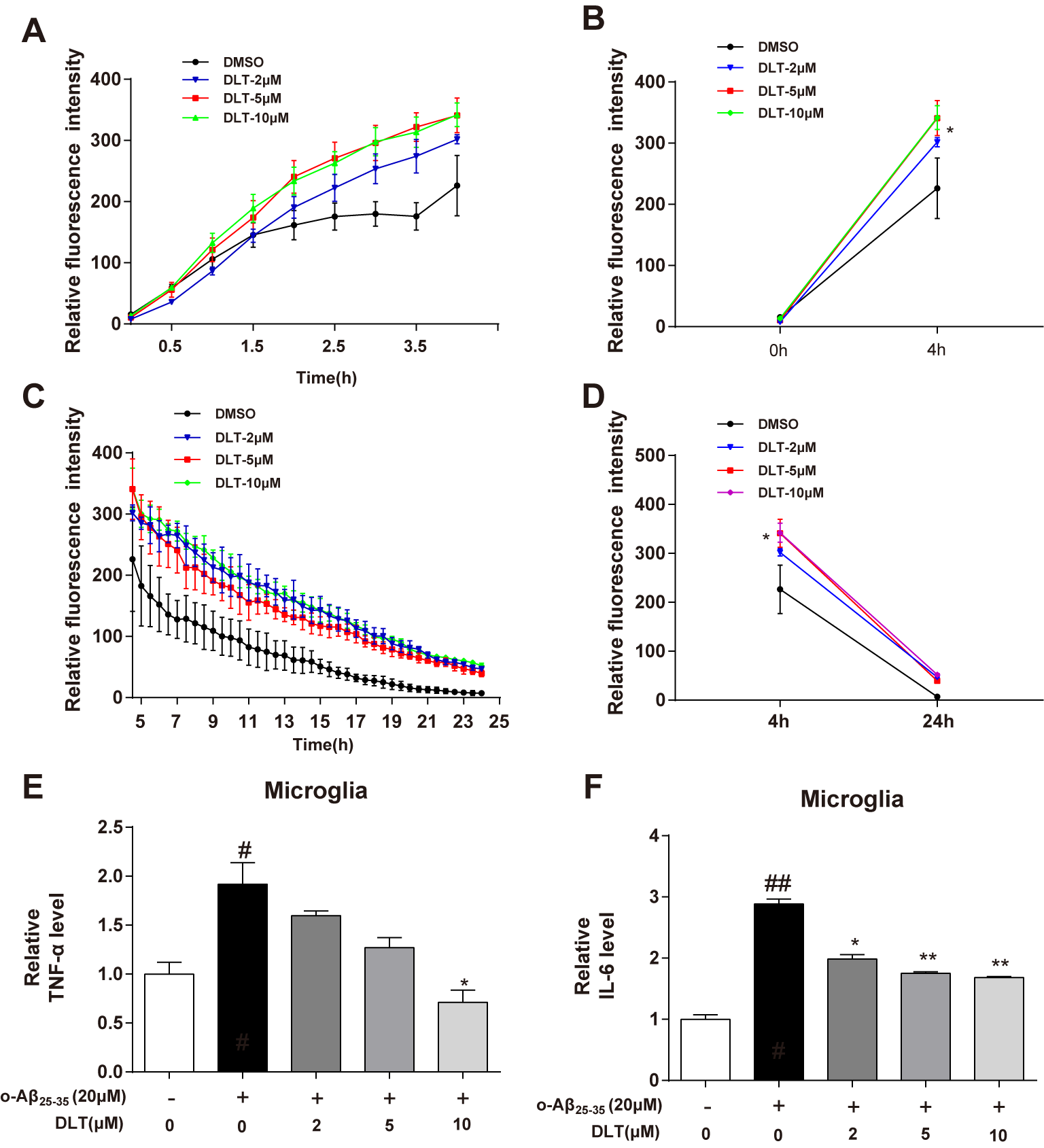

Supplement: Supplementary file 12 — Fig S11 [file ACEL-20-e13286-s012.tif]

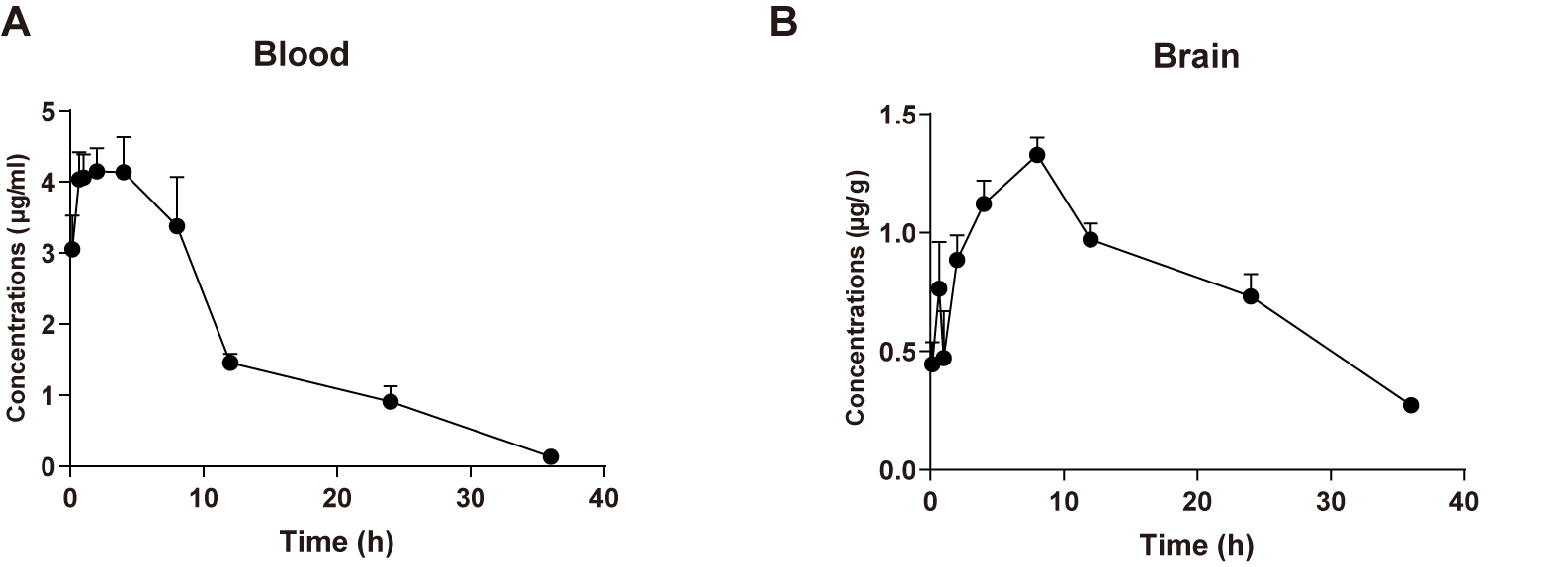

Supplement: Supplementary file 13 — Fig S12 [file ACEL-20-e13286-s013.tif]

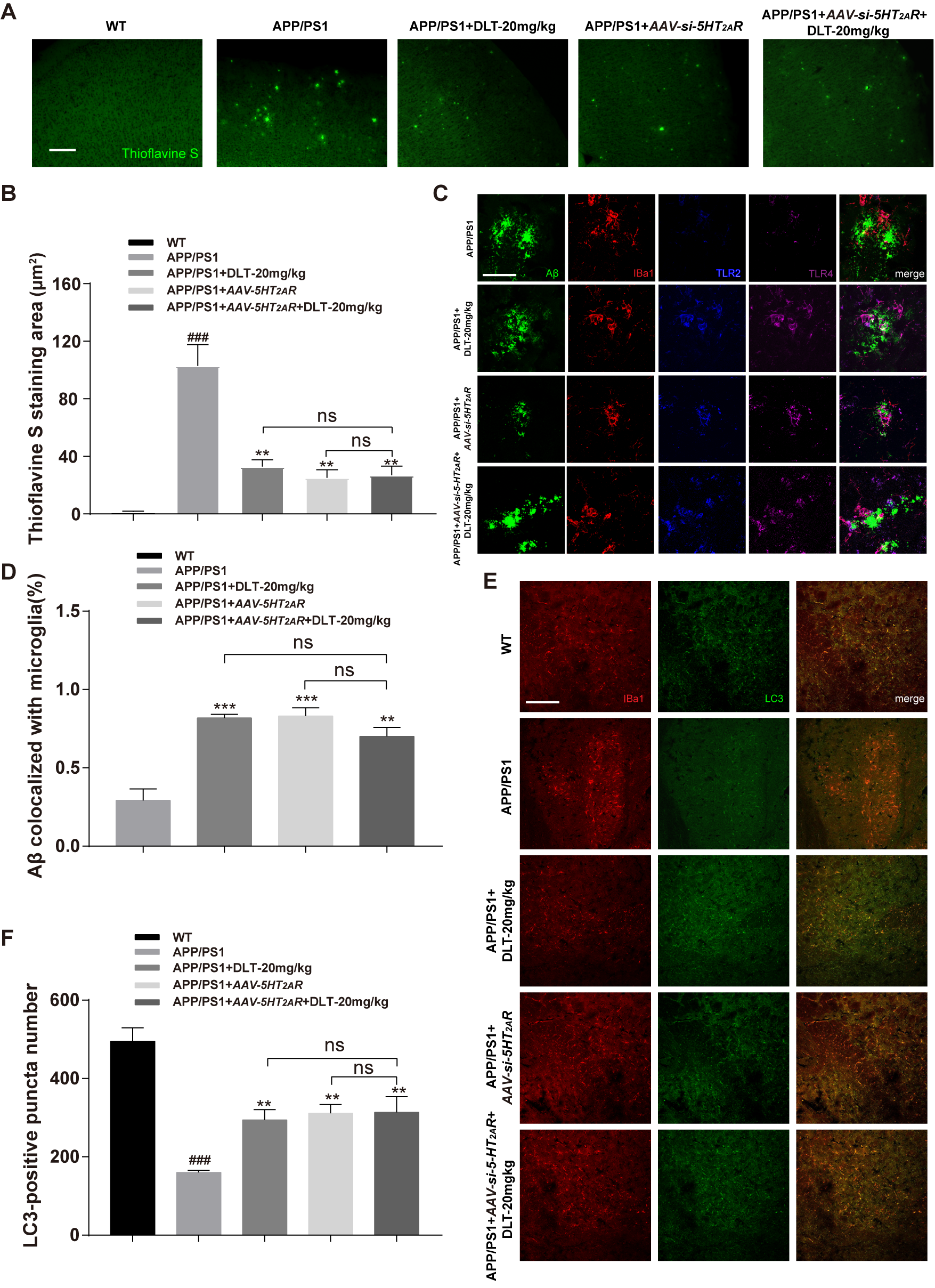

Supplement: Supplementary file 14 — Fig S13 [file ACEL-20-e13286-s014.tif]

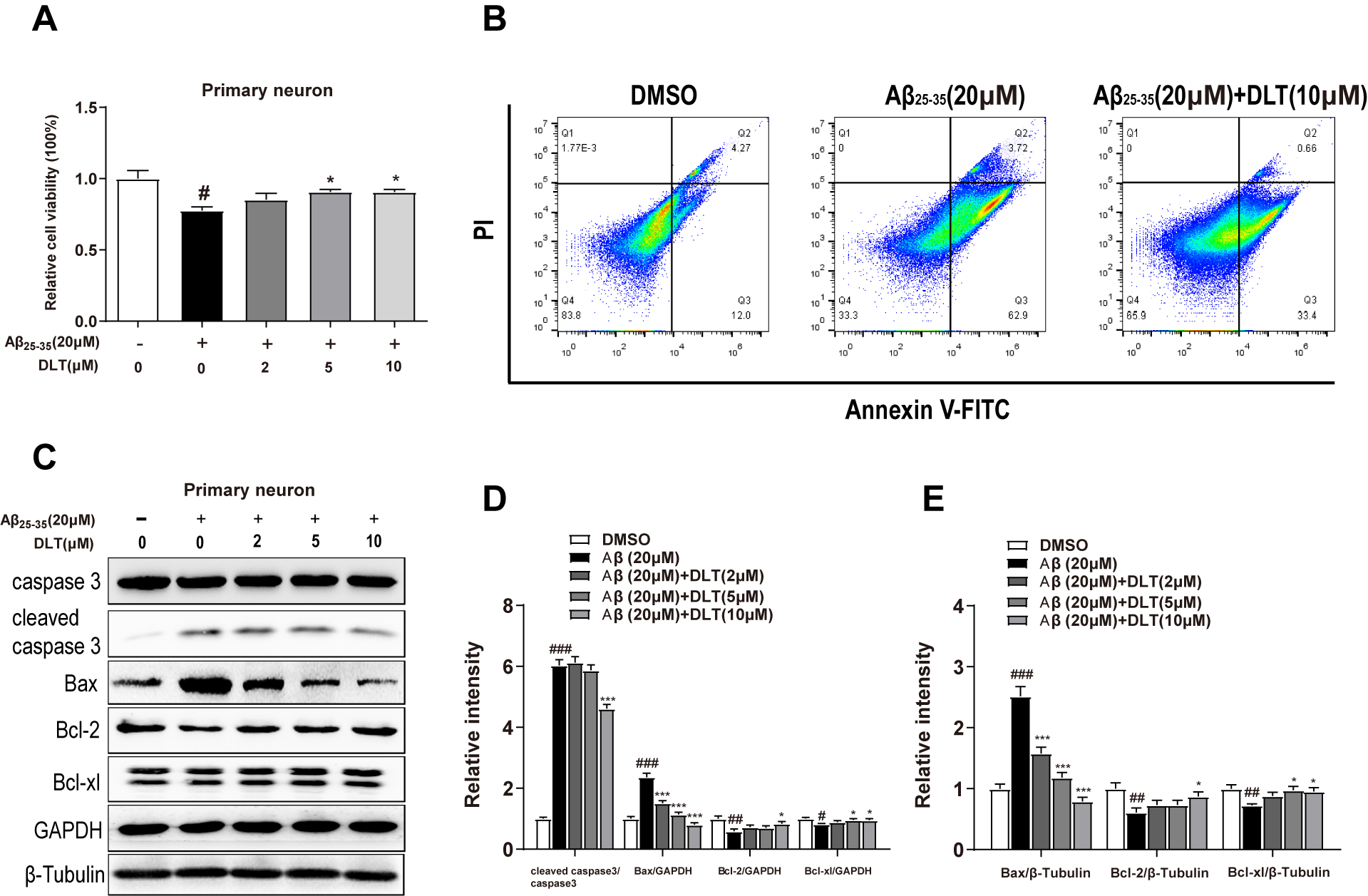

Supplement: Supplementary file 15 — Fig S14 [file ACEL-20-e13286-s015.tif]
